# The N6‐methyladenosine landscape of ovarian development and aging highlights the regulation by RNA stability and chromatin state

**DOI:** 10.1111/acel.14376

**Published:** 2024-10-15

**Authors:** Xiujuan Hu, Jiafeng Lu, Chenyue Ding, Jincheng Li, Qinyan Zou, Wenjuan Xia, Chunfeng Qian, Hong Li, Boxian Huang

**Affiliations:** ^1^ State Key Laboratory of Reproductive Medicine and Offspring Health, Suzhou Affiliated Hospital of Nanjing Medical University, Suzhou Municipal Hospital, Gusu School Nanjing Medical University Suzhou China

**Keywords:** autophagy, endogenous retroviruses, FTO, H3K9me3, METTL16, N6‐methyladenosine, ovarian aging, SUV39H1

## Abstract

The versatile epigenetic modification known as N6‐methyladenosine (m^6^A) has been demonstrated to be pivotal in numerous physiological and pathological contexts. Nonetheless, the precise regulatory mechanisms linking m^6^A to histone modifications and the involvement of transposable elements (TEs) in ovarian development and aging are still not completely understood. First, we discovered that m^6^A modifications are highly expressed during ovarian aging (OA), with significant contributions from decreased m^6^A demethylase FTO and overexpressed m^6^A methyltransferase METTL16. Then, using FTO knockout mouse model and KGN cell line, we also observed that FTO deletion and METTL16 overexpression significantly increased m^6^A levels. This led to the downregulation of the methyltransferase SUV39H1, resulting in reduced H3K9me3 expression. The downregulation of SUV39H1 and H3K9me3 primarily activated LTR7 and LTR12, subsequently activating ERV1. This resulted in a decrease in cell proliferation, while the levels of apoptosis, cellular aging markers, and autophagy markers significantly increased in OA. In summary, our study offers intriguing insights into the role of m^6^A in regulating DNA epigenetics, including H3K9me3 and TEs, as well as autophagy, thereby accelerating OA.

## Graphical abstract

The combination of FTO knockdown and METTL16 overexpression synergistically increased m6A levels, subsequently downregulating the expression of the H3K9me3 methyltransferase SUV39H1. This resulted in a reduction of H3K9me3 levels and the activation of the ERV1 family, notably LTR7 and LTR12. The activation of LTR7 and LTR12 led to a reduction in cell proliferation, along with a significant increase in apoptosis, cellular aging, and autophagy markers. These combined mechanisms collectively contribute to the advancement of ovarian aging.

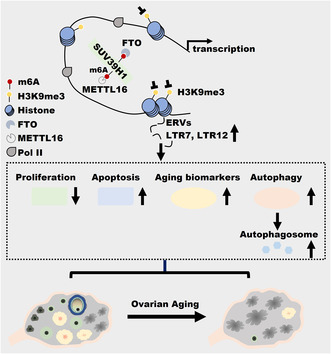

**Abbreviations**
ERVs: Endogenous retroviruses
EV: Empty vector
Fet: Fetal
FTO: Fat mass and obesity‐associated protein
H3K9me3: Histone H3 methylation at lysine 9
KD: Knockdown
KGN: Human ovarian granulosa cells
KO: Knockout
LTR12: Long Terminal Repeat 12
LTR7: Long Terminal Repeat 7
m^6^A: N6‐methyladenosine
MeRIP‐seq: m^6^A RNA immunoprecipitation sequencing
METTL16: Methyltransferase 16
Mop: Menopausal
OA: Ovarian aging
OD: Ovarian development
OE: Overexpressed
OFD: Ovarian function decline
SUV39H1: Suppressor of variegation 3‐9 homolog 1
TEs: Transposable elements
TESs: Transcription end sites
TSSs: Transcription start sites
Yng: Young

## 1 INTRODUCTION

The ovary is among the first organs to exhibit manifestations of senescence, typically occurring around the age of 35 in the human body, frequently resulting in age‐related infertility (Broekmans et al., 2009). Ovarian aging (OA) involves a gradual decline in oocyte quantity and quality, a substantial reduction in follicular reserves, and is a significant threat to female fertility (Burgess, 2021; Christensen et al., 2020). OA and development represent distinctive and relatively extreme physiological stages throughout the entire ovarian life cycle. A previous investigation pinpointed 290 genetic determinants that play a role in human OA (Ruth et al., 2021). Additionally, another study offered a comprehensive profile of pathogenic variations, encompassing 20 novel genes associated with the aging process of the ovaries (Ke et al., 2023). Interestingly, in the fetal ovary, approximately 80% of the initial oocyte pool is eliminated via the mysterious process of fetal oocyte attrition. Additionally, LINE1 activity initiates follicular atresia through apoptosis driven by DNA damage (Tharp et al., 2020). Furthermore, RUNX1 functions as a transcription factor implicated in the differentiation and maintenance of pregranulosa cells in the fetal ovary (Nicol et al., 2019). In addition, various factors, such as oxidative stress (Di Emidio et al., 2014), pyroptosis (Navarro‐Pando et al., 2021), and autophagy (Jin et al., 2022), can contribute to OA. For example, during oocyte maturation, autophagy in granulosa cells plays a beneficial role by selectively targeting ACLY to maintain a specific level of citrate (He et al., 2023). Furthermore, the dysregulation of mitophagy under the regulation of RAB7 can impact oocyte meiosis and subsequently accelerate OA (Jin et al., 2022). However, the upstream and downstream mechanisms of autophagy in OA have not been fully elucidated. Hence, an in‐depth comprehension of the complex regulatory mechanisms involved in OA is crucial for delaying OA, as it can reveal innovative drug targets and therapeutic strategies.

N6‐methyladenosine (m^6^A) represents the predominant modification found in various regions of diverse RNAs (Wang et al., 2014). Recently, accumulating evidence has revealed various biological functions of m^6^A, including its roles in determining mammalian oocyte competence (Ivanova et al., 2017), embryonic development (Batista et al., 2014), and regeneration (Weng et al., 2018). m^6^A is a dynamic and reversible modification catalyzed by methyltransferases such as the METTL3‐METTL14‐WTAP complex (Wang et al., 2016) or METTL16 (Pendleton et al., 2017) and erased by demethylases, including fat mass and obesity‐associated protein (FTO) (Jia et al., 2011). Notably, our recently published study demonstrated the critical involvement of FTO in the regulation of spermatogenesis in a m^6^A‐dependent manner (Wu, Li, et al., 2023). Meanwhile, the m^6^A methyltransferase METTL3 mitigates hMSC senescence by stabilizing the MIS12 through m^6^A‐dependent mechanisms (Wu et al., 2020). Furthermore, there exists a significant connection between aging and DNA epigenetics. Notably, the gradual reduction in H3K27ac levels can function as a predictive indicator of aging (Cheng et al., 2018). Moreover, CxxC‐finger protein 1 (CXXC1) is a key component of the network maintaining H3K4me3 marks, influencing oocyte aging in mice and humans (Wu et al., 2022). In addition to the association between histone modifications on euchromatin and aging, DNA histone modifications on heterochromatin and RNA epigenetics (m^6^A) are also intricately linked. For example, METTL3 can regulate H3K9me3 modification by recruiting the SETDB1/TRIM28 complex (Xu et al., 2021). DNA histone modifications are regulated by upstream enzymes, such as SUV420H2 and SUV39H1. Recently, a report indicated that H4K20me3 and SUV420H2 (the H4K20me3 methyltransferase) both play a critical role in silencing endogenous retroelements to prevent their activation during early development (Kurup et al., 2020). In addition, H3K9me3, which is associated with transcriptional repression, plays significant roles in suppressing mouse ERVs (Shilatifard, 2006). However, the mechanisms by which m^6^A regulates histone modifications, especially on heterochromatin, leading to OA have not been fully elucidated. Notably, m^6^A plays a role in maintaining cellular integrity by clearing reactive RNA species derived from endogenous retroviruses (ERVs) (Chelmicki et al., 2021). Therefore, we were interested in exploring whether m^6^A can influence OA through mechanisms involving ERVs.

During the process of evolution, mammalian genomes have accumulated a substantial number of transposable elements (TE). These elements can be broadly classified into two major categories: long terminal repeat (LTR) retrotransposons, which include ERVs, and non‐LTR retrotransposons, such as LINEs (Wicker et al., 2007). ERVs are integrated viral sequences in mammalian genomes, resulting from multiple ancient exogenous retrovirus invasions in germ‐line cells (Li et al., 2022), with significant effects on genome regulation and cellular physiology (Johnson, 2019). The relationship between m^6^A and retrotransposons in development and regeneration is gradually becoming clearer. For instance, FTO regulates LINE1 RNA through m^6^A demethylation, thereby influencing chromatin modification levels and the chromatin state in mESCs. Moreover, upon the activation of LTR7 (a subfamily of ERVs), it is regulated by the m^6^A reader YTHDC2 and exerts an inhibitory influence on the self‐renewal and regenerative capabilities of hESCs (Sun et al., 2023). While m^6^A's mechanisms in retrotransposon regulation during development are emerging, its role in retrotransposon regulation during OA remains unexplored. Excitingly, the inability to suppress particular ERVs, such as HERVK, has been associated with conditions such as infertility and tissue aging (Barau et al., 2016; Liu et al., 2023). This series of evidence has inspired our interest in whether m^6^A regulates histone modifications, thereby influencing ERVs and expediting OA. Hence, in this study, we primarily aimed to uncover how m^6^A downregulates the histone methyltransferase SUV39H1, diminishes H3K9me3 modification, and triggers the activation of ERVs, particularly LTR7 and LTR12, thereby leading to OA.

## 2 RESULTS

### 2.1 Overall differential m^6^A transcriptome profiling in follicular development and OA

**FIGURE 1:**
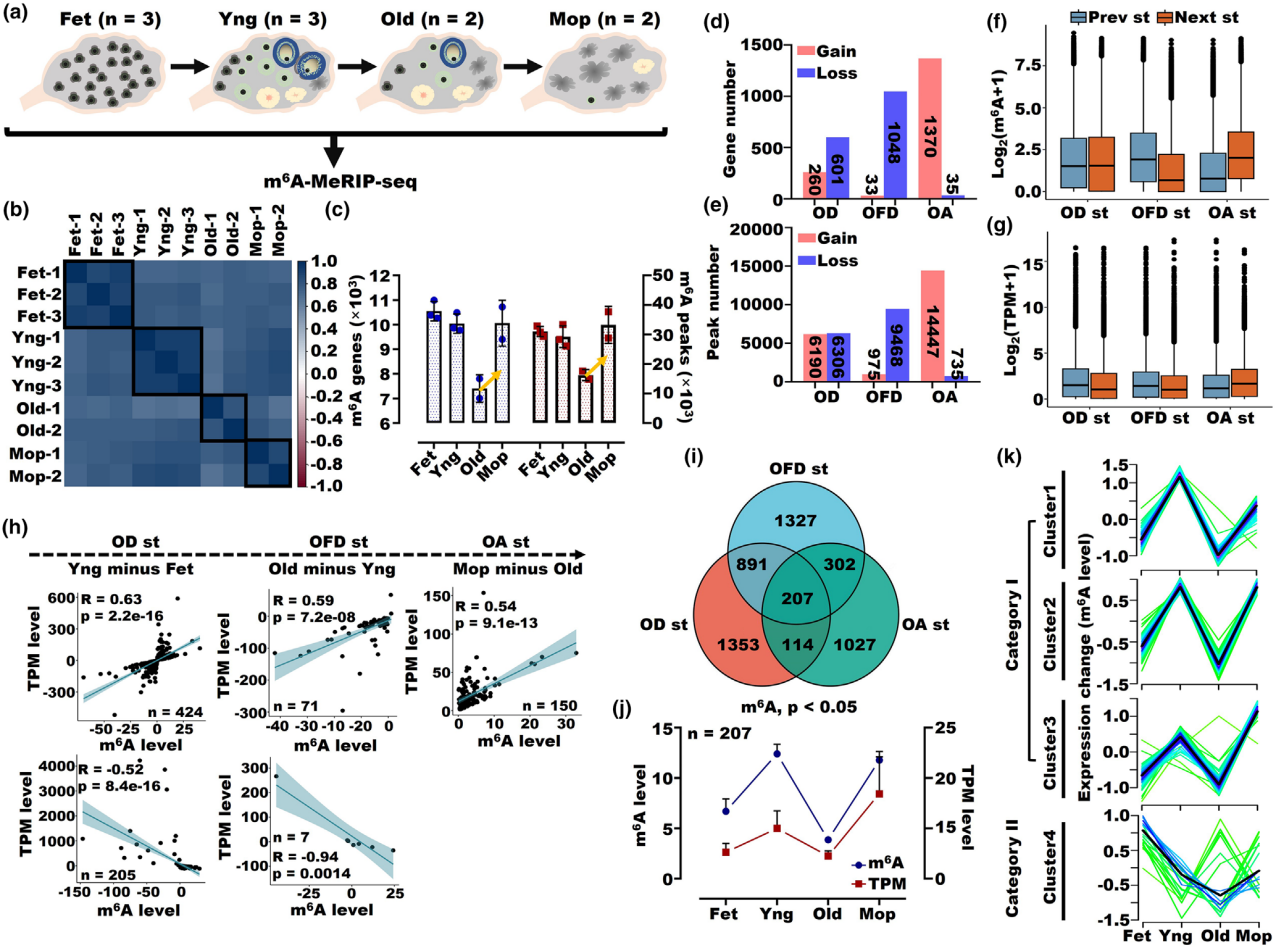
Overall differential transcriptome m^6^A profiling in different stages of human ovaries. (a) Schematic of the dynamic microstructure of the human ovary and m^6^A MeRIP sequencing. (b) Heatmap of Pearson correlation of the m^6^A levels of the matched samples between human ovaries. (c) The number of enriched m^6^A peaks and genes in human ovaries at different stages. (d) The number of genes with m^6^A gain and loss during ovarian development (OD, from Fet to Yng), ovarian function decline (OFD, from Yng to Old), and ovarian aging (OA, from Old to Mop). (e) The number of gain and loss m^6^A peaks during OD, OFD, and OA. (f) Box plot showing the expression levels of m^6^A genes during OD, OFD, and OA. (g) Box plot showing the mRNA expression levels during OD, OFD, and OA. (h) Coefficient correlation analysis of overlapping m^6^A and mRNA levels during OD, OFD, and OA (Fold change >2, next stage minus previous stage). (i) Venn diagram of the differentially expressed m^6^A genes in the OD, OFD and OA stages (*p* < 0.05). (j) Comparison of 207 genes with m6A and mRNA levels during the OD, OFD and OA stages. (k) Fuzzy c‐means clustering identified four various temporal patterns of m^6^A genes. The x‐axis represents four stages, while the y‐axis represents log_2_‐transformed, normalized intensity ratios in each stage.

To explore the temporal dynamics of m^6^A modifications throughout oocyte maturation and OA, we performed m^6^A‐MeRIP‐seq on 10 human ovarian samples, categorized into four stages: fetal (Fet), young (Yng), old (Old), and menopausal (Mop) (Figure 1a). Clustering analysis of m^6^A genes showed that m^6^A levels were more similar within the same group and more divergent between different groups (Figure 1b). Similarly, transcriptomic changes in ovarian samples followed a trend comparable to that of m^6^A levels (Figure S1a). The distribution pattern of m^6^A peaks was relatively uniform across all human ovarian samples, with a slight reduction in the proportion of intron m^6^A peaks in the Old group (Figure S1b). The average number of m^6^A peaks and genes significantly increased in the Mop group compared to the Old group (Figure 1c).

To delve deeper into the dynamics of m^6^A modification, we compared the m^6^A‐positive (m^6^A+) and m^6^A‐negative (m^6^A‐) transcripts and peaks across the four stages. The comparison revealed that the RNA expression levels of m^6^A+ transcripts were consistently higher than those of m^6^A‐ transcripts in all four stages (Figure S1c). Furthermore, we observed the presence of 4066, 3796, 1306, and 7941 unknown m^6^A peaks in the Fet, Yng, Old, and Mop groups, respectively, indicating a significant increase in unknown m^6^A peaks from the Old to Mop stage (Figure S1d). To offer a more in‐depth examination of the notable m^6^A changes observed between successive stages, we investigated the gain and loss of m^6^A genes or peaks during ovarian development (OD) from Fet to Yng, ovarian function decline (OFD) from Yng to Old, and OA from Old to Mop. The gain and loss of m^6^A genes and peaks exhibited a consistent trend, where most genes or peaks experienced m^6^A loss during the OFD stage, while the OA stage predominantly showed m^6^A gain, except for the OD stage, where the gain and loss events were relatively balanced (Figure 1d,e). In addition, we observed a significant decrease in the abundance of m^6^A‐modified genes in the Old group compared to the Yng group during the OFD stage. During the OA stage, the abundance of m^6^A‐modified genes and mRNA showed a significant increase in the Mop group compared to the Old group (Figure 1f,g). Additionally, the expression of m^6^A‐modified genes and mRNA exhibited a slight reduction during the OFD stage (Figure 1f,g). The results suggest a potential role of m^6^A in upregulating the expression levels of the transcriptome, particularly during the OA stage, thereby affecting transcription. Consistently, the analysis of m^6^A dot blots with RNA from mouse ovaries revealed a significant gradual increase in the global m^6^A level during mouse ovary aging (Figure S1e). Specifically, we observed that the expression of FTO protein exhibited a substantial decrease, while the expression of METTL16 protein demonstrated a noticeable increase during aging (Figure S1f,g). These results suggest that both FTO and METTL16 play a critical role in upregulating m^6^A modification during OA. Furthermore, our results also demonstrated a notable increase in the protein expression levels of both p27 and p53 in the aging process (Figure S1f,g).

In line with the above results, the imbalance of m^6^A gene regulation, comprising both upregulated and downregulated genes, remained relatively stable during the OD stage (Figure S2a,b). Significantly, there was a notable difference in the expression level of downregulated mRNA during the OD stage. (Figure S2b). Notably, the expression levels of m^6^A genes and mRNA were both significantly lower during the OFD stage (Figure S2c,d). Conversely, an opposite trend was observed in the Mop group, where an increase in the number of m^6^A genes and mRNA was evident during the OA stage (Figure S2e,f). Moreover, employing rigorous statistical analyses, we consistently observed a concordant trend in m^6^A gene and mRNA expression during both the OFD and OA stages, whereas an opposite trend was consistently observed during the OD stage. (Figure S2g–l). To obtain a comprehensive understanding of the gene expression patterns across adjacent stages, we performed a comparative analysis of m^6^A genes and mRNA, considering only those exhibiting a fold change >2 (Next stage minus previous stage). We further overlapped the differentially expressed m^6^A genes and mRNA (Fold change >2, next stage minus previous stage) between adjacent stages (Figure S2m–o). During the OD stage, we identified 1388 common genes, among which 424 and 205 genes exhibited moderate positive (*R* = 0.63, *p* < 0.001) and negative correlations (*R* = −0.52, *p* < 0.001) between m^6^A and mRNA expression levels, respectively (Figure 1h). Gene Ontology (GO) analysis revealed that both the histone modification and meiotic nuclear division pathway, were significantly associated with the 629 genes in the OD stage (Figure S2p). During the OFD stage, we observed 71 genes displaying a moderate positive correlation (*R* = 0.59, *p* < 0.001) and 7 genes exhibiting a strong negative correlation (*R* = −0.94, *p* < 0.001) between m^6^A gene and mRNA expression levels (Figure 1h). Moreover, we observed that protein kinase activity and the translation initiation pathway were enriched in the OFD stage (Figure S2q). Furthermore, during the OA stage, we observed a moderate positive correlation (*R* = 0.54, *p* < 0.001) among only 150 differentially expressed genes (Figure 1h). These differentially expressed genes were associated with mRNA catabolism and processing, chromatin remodeling, and histone modification processes, highlighting their relevance to the OA stage (Figure S2r).

Next, we performed an intersection analysis of the differentially expressed m^6^A genes in the OD, OFD and OA stages. As a result, we identified a total of 207 common m^6^A genes, thereby enabling us to evaluate their expression profiles across multiple stages (Figure 1i). Interestingly, we observed a striking trend among the expression profiles of these 207 common m^6^A genes across all stages. Specifically, both the m^6^A and mRNA expression levels of these genes exhibited a rapid increase in the OD stage, followed by a steep decline in the OFD stage and eventually a significant surge in the OA stage (Figure 1j).

The significant alterations in m^6^A gene expression during the OA process suggest that m^6^A involves in the regulation of ovarian senescence in humans. To further explore the temporal dynamics of m^6^A genes, we applied the fuzzy c‐means clustering algorithm to the 207 m^6^A genes that were commonly expressed across all stages. This approach revealed four distinct clusters of m^6^A gene expression patterns (Figure 1k). Notably, clusters 1, 2, and 3 (Category I) exhibited similar expression patterns as the dynamically regulated m^6^A genes, whereas cluster 4 (Category II) showed distinct expression patterns (Figure 1k). In addition, we also investigated the temporal dynamics of mRNA levels across the four identified clusters. Our findings indicated that clusters 1 and 2 displayed similar expression patterns as their corresponding m^6^A levels (Figure S3a,e). Our analysis revealed a notable difference between the Pearson's correlation coefficients of m^6^A levels and mRNA levels in category I (Figure S3b). To gain insight into the regulatory pathways of m^6^A modification in category I, we discovered that BPM4, TGFB2, and SOX5 were involved in crucial reproductive‐related pathways, including reproductive structure and reproductive development (Figure S3c), and noticed that both the m^6^A levels and mRNA levels of these three genes exhibited a similar trend to the temporal dynamics of category I (Figure S3d). We also determined the Pearson's correlation coefficients of m^6^A and mRNA levels in category II and found that while the majority of the genes showed a positive correlation between the two levels, a small number of genes exhibited an inverse correlation (Figure S3e,f). Furthermore, our GO analysis revealed that both the m^6^A and mRNA levels of FANCB exhibited a similar trend as those observed in category II and were involved in the DNA strand break repair pathway (Figure S3g,h).

### 2.2 M^6^A is highly enriched on ERVs during extreme ovarian senescence

**FIGURE 2:**
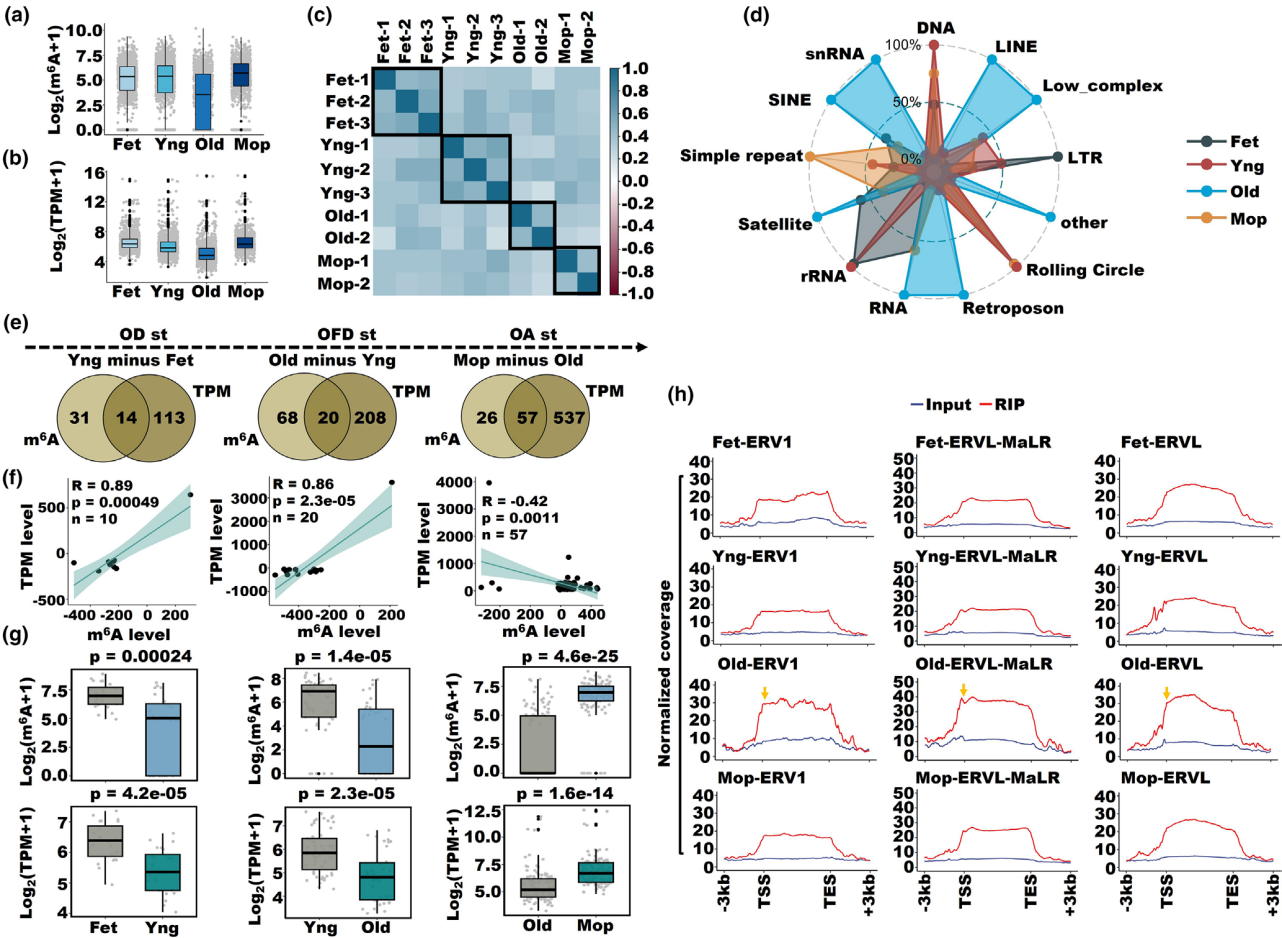
Global profile of m^6^A methylation and the transcriptome of retrotransposon RNA across four distinct stages. (a, b) The expression levels of m^6^A and the transcriptome of retrotransposon RNA in Fet, Yng, Old, and Mop. (c) Heatmap of Pearson correlation of the m^6^A levels of retrotransposon RNA in the matched samples between human ovaries. (d) The percentage of various retrotransposon RNA types with m^6^A modification in the four groups. (e) Venn diagram of the overlapping retrotransposon RNAs with and without m^6^A modification between adjacent stages (Next stage minus previous stage). (f) Correlation analysis between common retrotransposons with m^6^A and RNA expression levels during OD, OFD and OA. (g) Box plot showing the expression levels of m^6^A and RNA in retrotransposons during OD, OFD, and OA. (h) Average profile of m^6^A RIP and input signal of ERV1, ERVL‐MaLR and ERVL in Fet, Yng, Old, and Mop.

The derepression of certain retrotransposons, such as ERVs, has been implicated as a programmed mechanism in aging (Liu et al., 2023). Consequently, to examine the association between m^6^A methylation of retrotransposon RNA and OA, we quantified the global levels of m^6^A methylation and the transcriptome of retrotransposon RNA across four distinct stages (Figure 2a,b). In accordance with the previous trend of m^6^A‐modified mRNA, both the global m^6^A methylation and transcriptome levels of retrotransposon RNA exhibited a substantial decline during the OFD stage, followed by a pronounced increase in the OA stage (Figure 2a,b). Then, we conducted clustering analysis on the m^6^A levels of retrotransposon RNA across all samples, revealing greater similarity within the same group and increased divergence between different groups (Figure 2c). Considering the diverse types of retrotransposon RNA, we focused on LTR and LINE retrotransposon RNAs and performed clustering based on m^6^A levels. Notably, we observed significant intragroup similarity and substantial intergroup distinction among them (Figure S4a,b). Furthermore, we identified a noticeable decline in both the m^6^A and transcriptome levels of the majority of retrotransposon RNA types during the OD and OFD stages (Figure S4c,d). Interestingly, this declining trend was significantly reversed at the OA stage, as all listed retrotransposon RNAs exhibited a remarkable increase from the Old to Mop stages (Figure S4c,d). In particular, we calculated the percentage of various RNA types in the four groups and found a higher expression of LTR, LINE and SINE retrotransposons in the Old group (Figure 2d). Subsequently, we performed an overlap analysis of differentially expressed m^6^A‐modified retrotransposon RNAs (Fold change >2, the value from the next stage excludes the previous stage) between adjacent stages. In the OD stage, we identified 14 retrotransposons that were commonly expressed (Figure 2e). Among these, 10 retrotransposons exhibited a strong positive correlation (*R* = 0.89, *p* < 0.001) between m^6^A and RNA expression levels (Figure 2f). Similarly, during the OFD stage, we discovered 20 coexpressed retrotransposons that showed a significant positive correlation (*R* = 0.86, *p* < 0.001) between m^6^A and RNA expression levels (Figure 2f). Additionally, we observed that retrotransposon RNAs had significantly lower m^6^A and mRNA levels during the OD and OFD stages (Figure 2g). In contrast to the OD and OFD stages, the 57 retrotransposon RNAs that overlapped in the OA stage exhibited a negative correlation between m^6^A and mRNA levels and furthermore displayed a more prominent increase in both (Figure 2e–g). Moreover, during the transition of the OA stage, we observed a significant increase in both m^6^A and RNA expression levels for 27 LTR RNAs among the 57 retrotransposon RNAs (Figure S4e,f). Furthermore, we selected three representative RNA types from the ERV family: ERV1, ERV1‐MaLR, and ERVL. We observed consistent m^6^A expression of these three types throughout all stages of OD and senescence (Figure 2h). Notably, we observed a significant increase in m^6^A density from transcription start sites (TSSs) to transcription end sites (TESs), specifically in the Old group (Figure 2h). Furthermore, consistent with ERVs, L1, L2, and Alu displayed a similar pattern across all stages, particularly in the Old group (Figure S4g). These results highlight the prevalent m^6^A methylation on ERVs and other retrotransposon RNAs, indicating their potential contribution to OA.

### 2.3 FTO knockout triggers increased m^6^A modifications, impacting autophagic flux and OA

**FIGURE 3:**
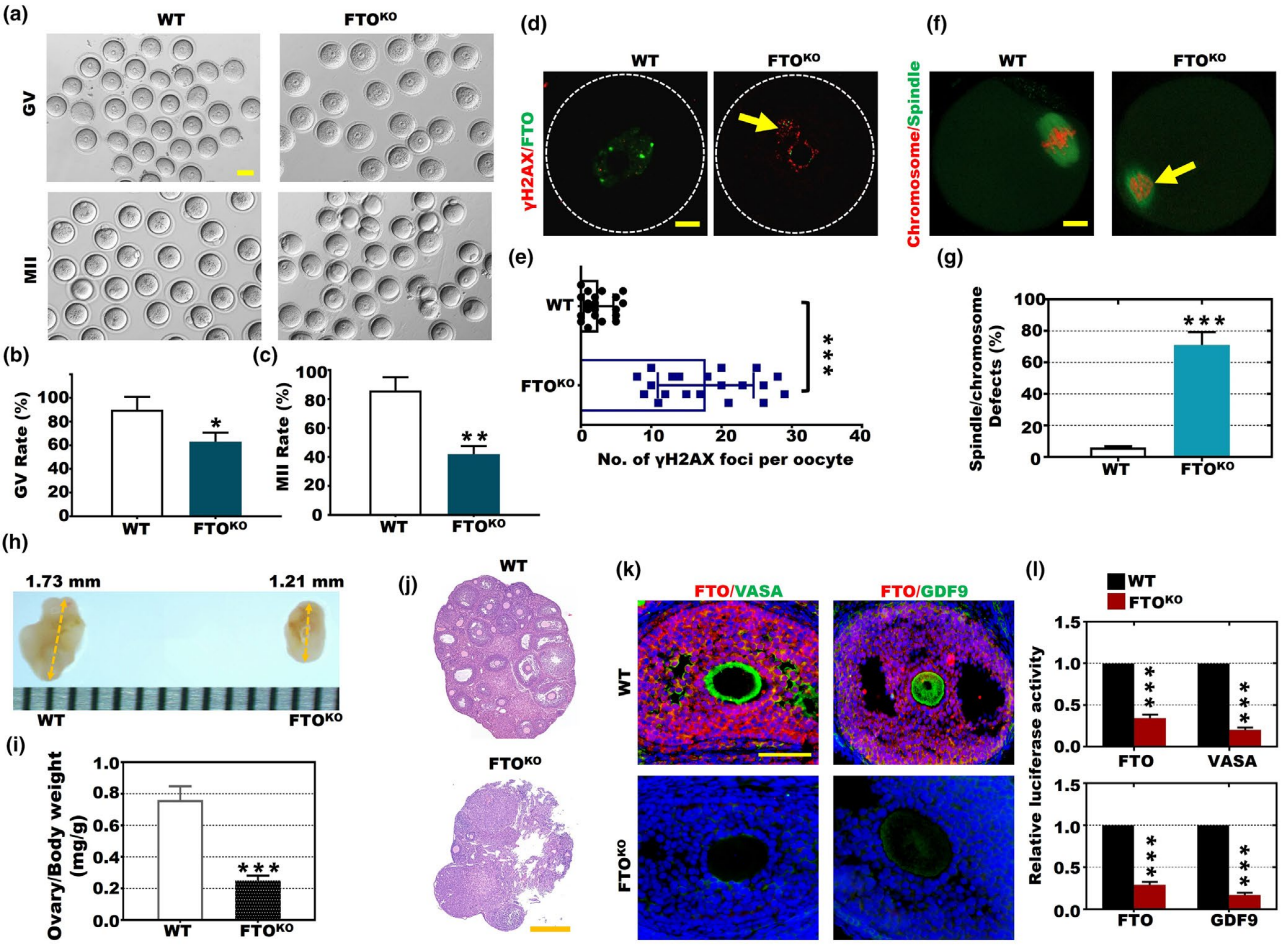
FTO^KO^ affected the developmental potential of oocytes. (a–c) The number of GV and MII oocytes obtained from FTO^KO^ and WT mice (Scale bars, 100 μm). (d) Images displaying γ‐H2AX (red) and FTO (green) staining in FTO^KO^ and WT oocytes. (e), The number of γ‐H2AX foci per oocyte in FTO^KO^ and WT oocytes (***p < 0.001). (f) Images displaying chromosome (red) and spindle (green) staining in FTO^KO^ and WT oocytes. (g) The percentage of spindle and chromosome defects in FTO^KO^ and WT oocytes (***p < 0.001). (h) The ovarian size in FTO^KO^ and WT mice. (i) The ratio of ovary weight to body weight in FTO^KO^ and WT mice (***p < 0.001). (j) HE staining of FTO^KO^ and WT ovaries (Scale bars, 500 μm). (k, l) Relative luciferase activity of FTO (red), VASA (green), and GDF9 (green) in the FTO^KO^ and WT groups (***p < 0.001, 50 μm).

First, immunofluorescence analysis revealed that FTO primarily localizes within the nucleus of the GV and the spindle during meiosis (Figure S5a). To substantiate that the high m^6^A level resulted in OA, we generated an FTO knockout (FTO^KO^) mouse model using CRISPR/Cas9, as described in previous literature (Wei et al., 2022; Wu, Li, et al., 2023) (Figure S5b). Conspicuously, a notable decrease in the protein expression of FTO was observed in both the oocytes and granular cells of FTO^KO^ mice (Figure S5c–e). FTO^KO^ mice exhibited more fewer pups per litter compared to wild‐type (WT) mice (Figure S5f,g), with an age‐related decline particularly observed between 20 and 26 weeks (Figure S5h). Furthermore, 6‐month‐old FTO^KO^ mice displayed a consistent reduction in the number of oocytes obtained at both the GV and MII stages compared to WT (Figure 3a–c). To investigate the impact of FTO on DNA damage during oocyte development, immunofluorescence (IF) analysis was performed and demonstrated a significant elevation in the number of γ‐H2AX foci per oocyte in FTO^KO^ mice in comparison to WT (Figure 3d,e). Additionally, 6‐month‐old FTO^KO^ oocytes displayed a higher incidence of spindle and chromosome defects than WT (Figure 3f,g). Furthermore, 6‐month‐old FTO^KO^ mice exhibited significantly smaller ovary dimensions (1.21 mm) and a lower ratio of ovary weight to body weight than WT (Figure 3h,i). Histological examination using HE staining revealed a notable decrease in the number of follicles in the 6‐month‐old FTO^KO^ ovary compared to the WT ovary (Figure 3j), indicating impaired follicular development in the absence of FTO. Subsequently, we assessed the ovaries of 12‐month‐old FTO^KO^ and WT mice and observed a marked decrease in the number of follicles in the FTO^KO^ ovary compared to the WT ovary (Figure S6a,b). The expression of VASA and GDF9 in the ovary is essential for proper oocyte development and follicle formation. Hence, by conducting immunofluorescence (IF) analysis, we not only observed a notable decrease in the luciferase activity of FTO, VASA, and GDF9 in 6‐month‐old FTO^KO^ oocytes compared to WT (Figure 3k,l) but also witnessed a further decline in the luciferase activity of FTO, VASA, and GDF9 in 12‐month‐old FTO^KO^ oocytes relative to WT (Figure S6c–f).

To investigate the correlation between autophagic flux and OA, we performed western blotting to examine the expression levels of autophagy‐related proteins in the ovaries of 6‐month‐old FTO^KO^ and WT ovary (Figure S7a). We initially observed a significant reduction in the protein levels of p62 and four members of the ATG family (ATG3, ATG5, ATG7, ATG12) in the ovaries of 6‐month‐old FTO^KO^ mice (Figure S7b–f). Conversely, the protein levels of the aging‐associated proteins p27 and p53 displayed an inverse trend (Figure S7g,h). Moreover, we observed a significant increase in the ratio of LC3B II to LC3B I in the 6‐month‐old FTO^KO^ ovary (Figure S7i–j), indicating elevated autophagic flux. Additionally, electron microscopy analysis revealed a marked increase in the number of autophagosomes in both of 6‐month‐old and 12‐month‐old FTO^KO^ ovaries (Figure S7k,l). Therefore, FTO knockout can result in dysregulated autophagic flux, which may impact normal ovarian function and contribute to the onset of aging.

### 2.4 FTO knockout induced apoptosis and modulated the m^6^A levels of retrotransposon RNAs

**FIGURE 4:**
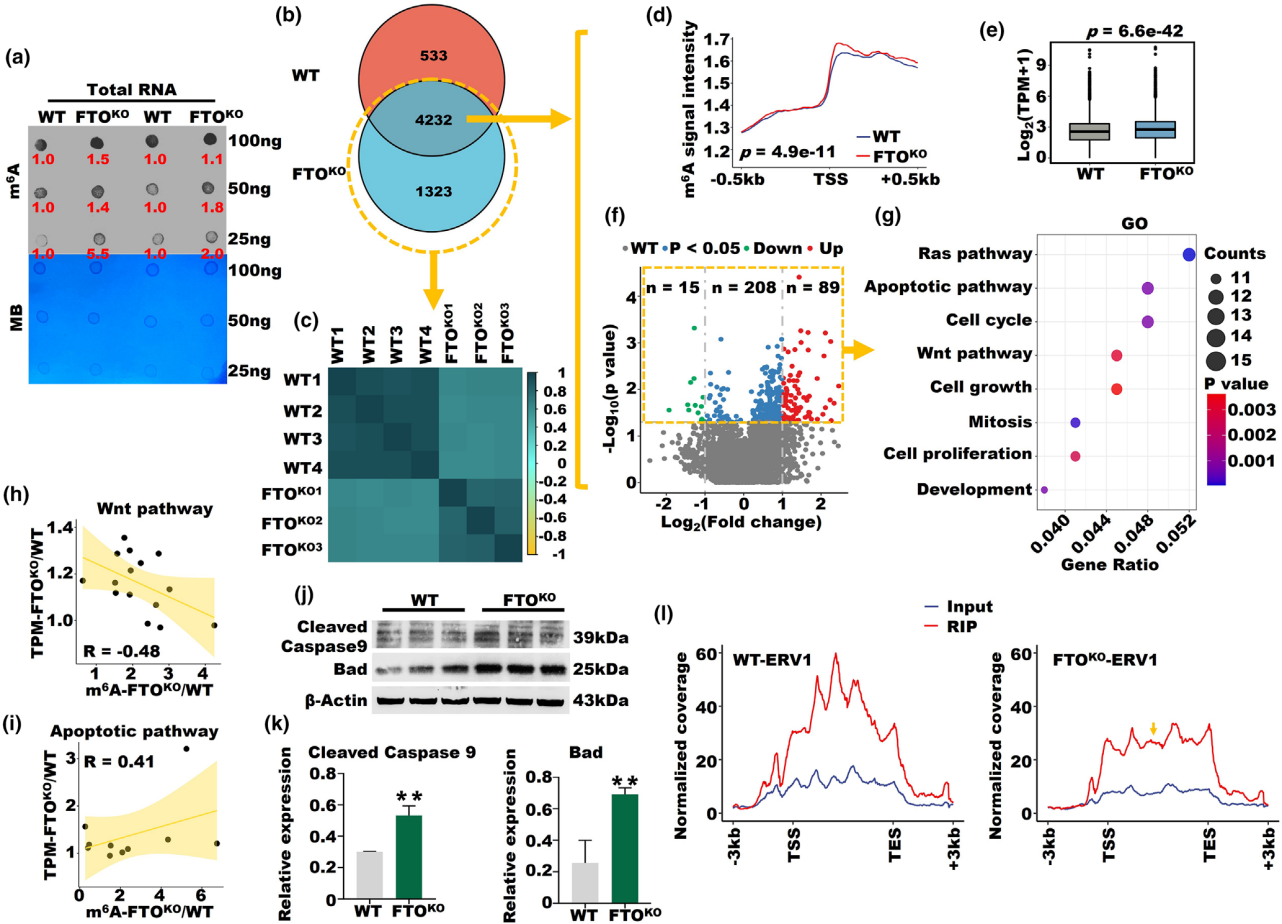
FTO^KO^ triggered apoptosis and influenced the m^6^A levels of retrotransposon RNAs. (a) The m^6^A dot blot of total RNA samples from the ovaries of six‐month‐old FTO^KO^ and WT mice. (b) Venn diagram of the m^6^A‐modified genes between the FTO^KO^ and WT mice. (c) Heatmap displaying the Pearson correlation of m^6^A‐modified genes exclusively found in the FTO^KO^ group compared to the WT mice. (d) The m^6^A signal intensity of the 4232 overlapping genes at the promoter regions in FTO^KO^ and WT mice. (e) Box plot showing the mRNA expression levels of 4232 overlapping genes in FTO^KO^ and WT mice. (f) Volcano plot showing the differentially expressed m^6^A genes in FTO^KO^ and WT mice. The x‐axis represents the log_2_‐transformed fold change, while the y‐axis represents the log_10_ normalized *p* value. (g) GO analysis of 312 differentially expressed m^6^A‐modified genes. (h, i) Pearson's correlation coefficients of m^6^A and mRNA levels in the Wnt and apoptotic signaling pathways. (j, k) Protein expression of cleaved Caspase 9 and Bad in FTO^KO^ and WT mice (*p < 0.05, ***p < 0.001). (l) Average profile of m^6^A RIP and input signal of ERV1 in FTO^KO^ and WT mice.

To elucidate the potential involvement of FTO in the regulation of crucial pathways and retrotransposon RNAs associated with OA, we explored the relationship between differentiated m^6^A‐modified RNA after FTO knockout and OA. Initially, the FTO knockout in the six‐month‐old ovaries resulted in a notable augmentation in the overall abundance of m^6^A modifications in total RNA samples at different concentrations (Figure 4a). Consistently, both m^6^A peaks and genes showed a slight increase in the FTO^KO^ group compared to the WT (Figure S8a,b). Furthermore, we observed a statistically significant increase in the signal intensity of m^6^A‐modified genes at the promoter regions (−0.5 kb and + 0.5 kb around the transcription start site, TSS) after FTO knockout (Figure S8c). Additionally, a marked increase in the expression levels of m^6^A‐modified genes and mRNA was observed after FTO knockout (Figure S8d,e). Subsequently, we identified 4232 overlapping m^6^A‐modified genes between the FTO^KO^ and WT groups and 1323 and 533 unique m^6^A‐modified genes in the FTO^KO^ and WT groups, respectively, with statistical significance set at *p* < 0.05 (Figure 4b). Additionally, the FTO^KO^ group exhibited 1323 unique m^6^A‐modified genes, while the WT group had 533 unique m^6^A‐modified genes (Figure 4b). Notably, it is worth noting that the overall m^6^A levels of 5555 m^6^A‐modified genes belonging to the FTO^KO^ group exhibited a higher degree of similarity within the same group, while displaying a more pronounced divergence when compared to different groups (Figure 4c). Moreover, upon depletion of FTO, there was a significant change in the m^6^A signal intensity of the 4232 overlapping genes at the promoter regions (−0.5 kb and + 0.5 kb around TSS) (Figure 4d). Importantly, FTO knockout resulted in a pronounced elevation in the transcriptome level, suggesting a substantial impact on gene expression (Figure 4e). Consistently, the expression levels of m^6^A‐modified genes exhibited a significant increase upon FTO knockout (Figure 4f). Building upon these findings, we conducted GO analysis on a subset of 312 significantly differentially expressed m^6^A‐modified genes (*p* < 0.05) and demonstrated that these m^6^A‐modified genes were associated with several crucial biological pathways, including the wnt signaling and apoptotic pathways (Figure 4g). Remarkably, the genes involved in the wnt signaling pathway and those associated with the apoptotic pathway exhibited moderate negative correlations (*R* = −0.48) and positive moderate correlations (R = 0.41) between the m^6^A modification and mRNA expression levels, respectively (Figure 4h,i). Furthermore, the validity of our findings was strengthened by the significant upregulation in the protein levels of two key effector caspases involved in apoptotic pathways, namely, cleaved Caspase 9 and Bad, after FTO^KO^ (Figure 4j,k).

Furthermore, we made an intriguing observation regarding the global m^6^A methylation levels of retrotransposon RNA, which exhibited a significant increase after FTO knockout (Figure S8f). Specifically, there was a pronounced elevation in the m^6^A expression levels of three retrotransposon RNA types, namely, LTR, simple repeat, and SINE, following FTO knockout (Figure S8g). Notably, within the FTO^KO^ group, we observed a noteworthy reduction in m^6^A density specifically within the intermediate region between the TSS and TES of ERV1 (Figure 4l). In contrast, there was a significant increase in m^6^A density near the TES of ERVL, while a notable reduction was observed within the intermediate region of ERVL‐MaLR (Figure S8h). Interestingly, the m^6^A density of ERVK remained unaffected by FTO knockout (Figure S8h). Additionally, we revealed a simultaneous increase in the m^6^A levels of SUV39H1, a writer responsible for the heterochromatin‐associated histone mark H3K9me3, and Hdac6, an eraser of the active histone mark H3K27ac, within the FTO^KO^ group (Figure S8i–l). In conclusion, our study revealed that FTO knockout exerted a regulatory influence on the inhibition of wnt and activation of apoptotic pathways, modulation of retrotransposon RNA m^6^A levels, and subsequent alteration of gene expression and pathways associated with OA.

### 2.5 FTO knockdown modulated the SUV39H1‐H3K9me3‐ERV axis in OA

**FIGURE 5:**
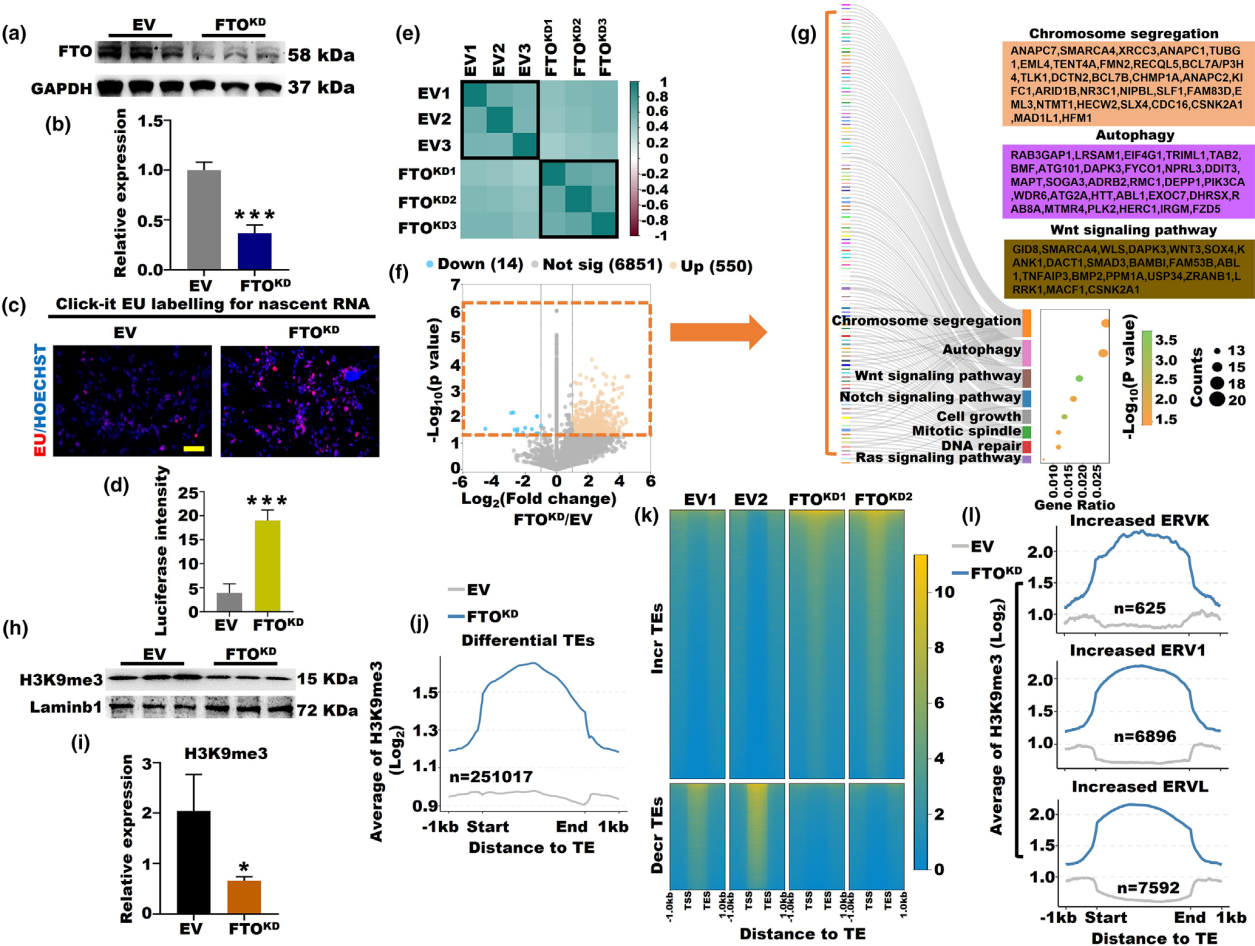
FTO^KD^ influenced the SUV39H1‐H3K9me3‐ERV axis during ovarian aging. (a, b) Protein expression of FTO in the FTO^KD^ and EV KGN cell lines (***p < 0.001). (c, d) The luciferase intensity signal of nascent RNA in the FTO^KD^ and EV groups (***p < 0.001). (e) Heatmap displaying the Pearson correlation of m^6^A‐modified genes in the FTO^KD^ and EV groups. (f) Scatterplots showing the differentially expressed m^6^A genes in the FTO^KD^ and EV groups. The x‐axis represents the log_2_‐transformed fold change, while the y‐axis represents the log_10_‐normalized *p* value. (g) GO analysis of differentially expressed m^6^A‐modified genes in the FTO^KD^ and EV groups. (h, i) The protein expression of H3K9me3 in the FTO^KD^ and EV KGN cell lines (*p < 0.05). (j) Average H3K9me3 signal on differential TEs in the FTO^KD^ and EV groups. (k) H3K9me3 signals on increased and decreased TEs in the FTO^KD^ and EV groups. (l) Average H3K9me3 signal on the increased ERVK, ERV1, and ERVL in the FTO^KD^ and EV groups. EV, empty vector.

To deepen our comprehension and solidify the central role of elevated m^6^A levels due to reduced FTO in OA, we generated a KGN cell line with FTO knockdown (FTO^KD^) (Figure 5a,b). Evidently, the FTO^KD^ cell line prominently displayed a notable augmentation in overall m^6^A modifications across various concentrations in total RNA samples (Figure S9a,b). Remarkably, a significantly higher luciferase intensity signal originating from nascent RNA was evident in the FTO^KD^ group than in the EV (Empty vector) group (Figure 5c,d). As expected, the FTO^KD^ group exhibited a marked surge in the number of m^6^A peaks and m^6^A genes, accompanied by heightened m^6^A levels (Figure S9c–e). Notably, m^6^A‐modified genes exhibited strong similarity within both the FTO^KD^ and EV groups but exhibited marked divergence across different groups (Figure 5e). Additionally, we observed a substantial predominance of upregulated m^6^A‐modified genes in the FTO^KD^ group, outnumbering the downregulated genes by approximately 39‐fold (Figure 5f). Consequently, we conducted GO analysis on a subset of significantly differentially expressed m^6^A‐modified genes (*p* < 0.05), revealing their strong correlation with eight key biological pathways, encompassing chromosome segregation, autophagy, and wnt signaling pathway (Figure 5g). In line with this, we noted a significant increase in the protein expression levels of autophagy‐related markers, specifically LC3B and p62, in the FTO^KD^ group (Figure S9f–i). Moreover, the expression of the aging‐induced proteins p21 and p53 was notably increased in the FTO^KD^ group compared to the EV (Figure S9j–l). Interestingly, the FTO^KD^ group exhibited decreased H3K9me3 protein expression compared to the EV (Figure 5h,i). Additionally, a decrease in SUV39H1 (H3K9me3 methyltransferase) expression was observed in the FTO^KD^ group compared to the EV (Figure S9m,n). Collectively, these findings provide deep insights into the vital contribution of elevated m^6^A levels due to FTO knockdown in the process of OA.

In continuation of our study, we overexpressed FTO in the KGN cell line while introducing mutations into the overexpressed FTO plasmid, resulting in FTO^OE^ and FTO^MUT^ variants as reported previously (Jia et al., 2011) (Figure S10a,b). Moreover, the overall m^6^A level in the FTO^MUT^ group was restored to that of the EV, indicating the loss of FTO^MUT^'s ability to decrease m^6^A levels (Figure S10c,d). We also noticed that the H3K9me3 protein expression of FTO^MUT^ was reduced to that of EV compared to that of FTO^OE^. We also observed a decrease in H3K9me3 protein expression in the FTO^MUT^ group compared to the FTO^OE^ group, similar to the expression levels in the EV group (Figure S10e,f). To further explain whether FTO regulates m^6^A modification to impact histones, we proceeded to compare the H3K9me3 signals using cut & tag sequencing and observed a dearth of H3K9me3 peaks on p21 and p53 in the FTO^KD^ group in comparison with the EV (Figure S10g,h), a trend that aligns well with the western blot results of p21 and p53 (Figure S9j–l). Additionally, we identified a significant increase in the abundance of differential TEs within the H3K9me3 signals in the FTO^KD^ group compared to the EV (Figure 5j). Subsequently, we conducted analyses of H3K9me3 signals on these differential TEs in both the FTO^KD^ and EV groups, revealing strikingly heightened H3K9me3 signals specifically on the increased TEs within the FTO^KD^ group (Figure 5k). Furthermore, we identified heightened H3K9me3 signals on the increased ERVK, ERV1, and ERVL retrotransposons within the FTO^KD^ group in contrast to the EV group (Figure 5l), with the number of increased ERVK, ERV1, and ERVL retrotransposons being approximately 8‐fold, 4‐fold, and 3‐fold higher, respectively, compared to the decreased ERV1 and ERVL retrotransposons in the FTO^KD^ group (Figure S10i). Compared to LTR retrotransposons, the non‐LTR retrotransposons showed a smaller increase in quantity. Specifically, the count of increased L1 and L2 retrotransposons in the FTO^KD^ group was approximately two times higher than that of the decreased L1 and L2 retrotransposons, while the difference was threefold for Alu retrotransposons (Figure S10j). In summary, our study revealed that FTO knockdown influences SUV39H1 and subsequently impacts the regulation of H3K9me3 and downstream ERV elements through m^6^A modifications, unveiling intricate connections among these factors in the context of OA.

### 2.6 METTL16 overexpression mediates the SUV39H1‐H3K9me3‐ERV axis in OA

**FIGURE 6:**
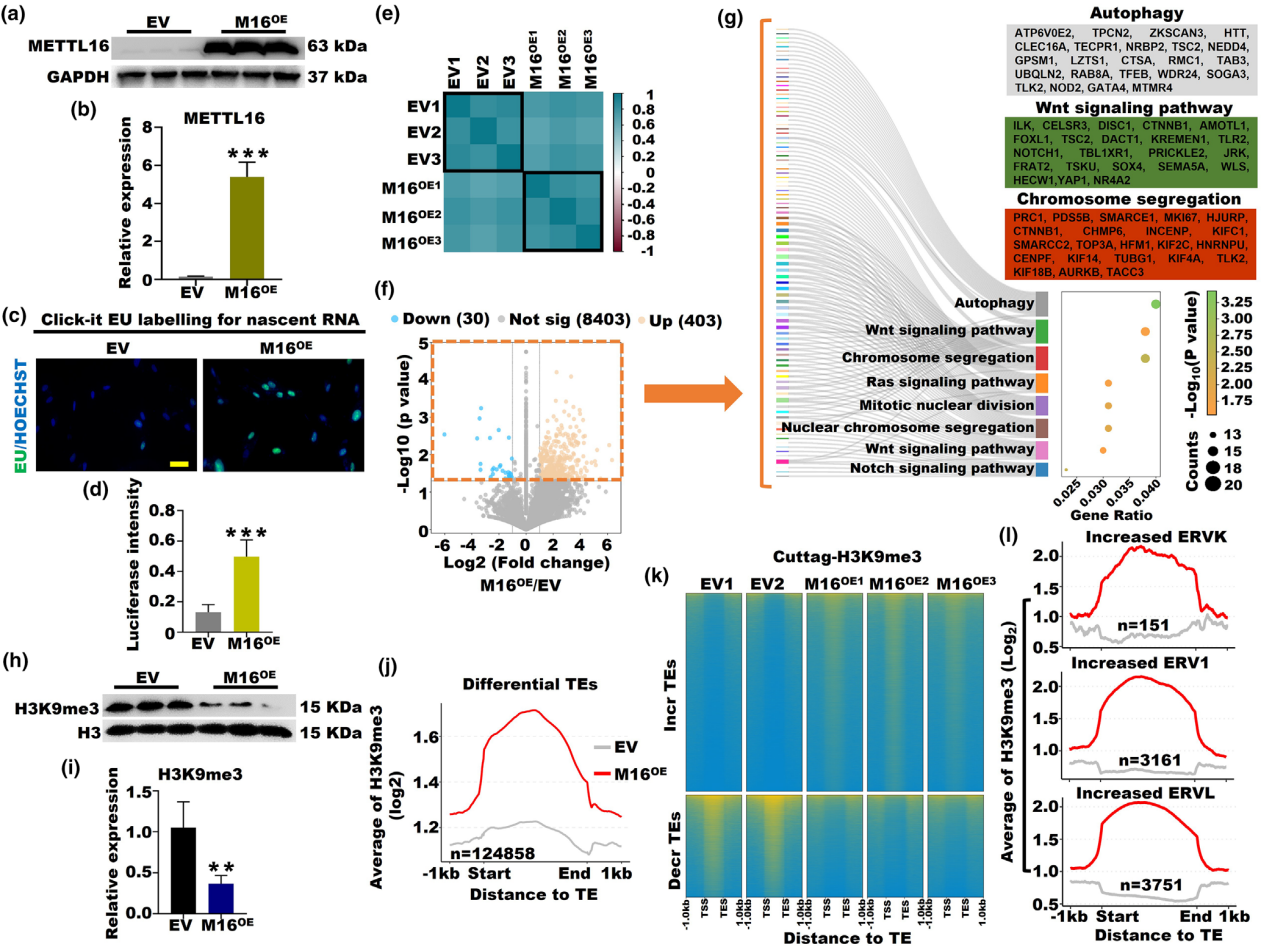
METTL16 overexpression regulated the SUV39H1‐H3K9me3‐ERVs axis in ovarian aging. (a, b) The protein expression of METTL16 in the M16^OE^ and EV KGN cell lines (***p < 0.001). (c, d) The luciferase intensity signal of nascent RNA in the M16^OE^ and EV groups (***p < 0.001). (e) Heatmap displaying the Pearson correlation of m^6^A‐modified genes in the M16^OE^ and EV groups. (f) Scatterplots showing the differentially expressed m^6^A genes in the M16^OE^ and WT groups. The x‐axis represents the log_2_‐transformed fold change, while the y‐axis represents the log_10_‐normalized *p* value. (g) GO analysis of differentially expressed m^6^A‐modified genes in the M16^OE^ and EV groups. (h, i) The protein expression of H3K9me3 in the M16^OE^ and EV KGN cell lines (**p < 0.01). (j) Average H3K9me3 signal on differential TEs in the M16^OE^ and EV groups. (k) H3K9me3 signals on increased and decreased TEs in the M16^OE^ and EV roups. (l) Average H3K9me3 signal on the increased ERVK, ERV1, and ERVL in the M16^OE^ and EV groups.

To provide comprehensive evidence supporting the involvement of high m^6^A levels caused by m^6^A methyltransferase in OA, we developed a KGN cell line with METTL16 overexpression (M16^OE^). Evidently, the M16^OE^ cell line exhibited a remarkable increase in METTL16 protein expression (Figure 6a,b) and a significant increase in overall m^6^A modifications in total RNA samples (Figure S11a,b). Fascinatingly, we noticed a significantly higher luciferase intensity signal from nascent RNA in the M16^OE^ group than in the EV (Figure 6c,d). As anticipated, the M16^OE^ group showed a slight increase in the number of m^6^A peaks and m^6^A genes, along with elevated m^6^A levels (Figure S11c–e). Significantly, m^6^A‐modified genes within the M16^OE^ and EV groups exhibited high similarity within each group but pronounced divergence between different groups (Figure 6e). Subsequently, we observed a substantial prevalence of upregulated m^6^A‐modified genes in the M16^OE^ group, significantly outnumbering the downregulated counterparts (Figure 6f). To further explore the biological significance, we performed Gene Ontology analysis on a subset of significantly differentially expressed m^6^A‐modified genes (*p* < 0.05) and found their strong association with eight important biological pathways, including autophagy, the wnt signaling pathway, and chromosome segregation (Figure 6g). Consistently, we observed a notable increase in the protein levels of autophagy‐related markers, including p62 and LC3B, in the M16^OE^ group (Figure S11f–h). Additionally, we detected upregulation of the expression of the proapoptotic protein BIM and downregulation of the expression of the antiapoptotic protein BCL‐2 in the M16^OE^ group compared to the EV (Figure S11i–k). These findings offer profound insights into the crucial role of high m^6^A levels induced by METTL16 overexpression in the OA process.

Previous studies have indicated that the methyltransferase complex METTL3/METTL14 regulates H3K9me2 modification (Li et al., 2020). Intriguingly, in the M16^OE^ group, we observed a significant decrease in the protein expression of H3K9me3 compared to that in the EV (Figure 6h,i). Furthermore, we conducted a comparison of the H3K9me3 signals using Cut & Tag sequencing and observed a substantial increase in the abundance of differential TE within the H3K9me3 signals in the M16^OE^ group compared to the EV (Figure 6j). We then conducted individual analyses of H3K9me3 signals on differential TEs in both the M16^OE^ and EV groups. Remarkably, we observed heightened H3K9me3 signals on the increased TEs in the M16^OE^ group (Figure 6k). Concurrently, we observed a scarcity of H3K9me3 peaks on the aging‐related marker p38 and the autophagy‐related marker p62 in the M16^OE^ group compared with the EV (Figure S11l–m), in line with the western blot results for p38 and p62 (Figures S11f,g and Figure S12a,b). Moreover, we noticed an upregulation in the expression of both p38 and the aging‐related protein p16, along with a downregulation of SUV39H1, in the M16^OE^ group compared to the EV (Figure S12a,b). Notably, we detected elevated H3K9me3 signals on the increased ERVK, ERV1, and ERVL retrotransposons in the M16^OE^ group compared to the EV (Figure 6l). Meanwhile, the count of increased ERV1 and ERVL retrotransposons was approximately three times higher than that of the decreased ERV1 and ERVL retrotransposons in the M16^OE^ group (Figure S12c). Expanding beyond the LTR family, we noted a comparable pattern within the non‐LTR family. To be precise, the quantity of increased LINE2 and Alu retrotransposons was roughly twice as high as the count of decreased LINE2 and Alu retrotransposons in the M16^OE^ group (Figure S12d). In conclusion, our discoveries offer deep insights into the complex interplay involving m^6^A modifications, H3K9me3, and ERV1 and ERVL retrotransposons within the context of OA in the M16^OE^ cell line.

### 2.7 High m^6^A methylation‐induced activation of ERV1 triggers OA

**FIGURE 7:**
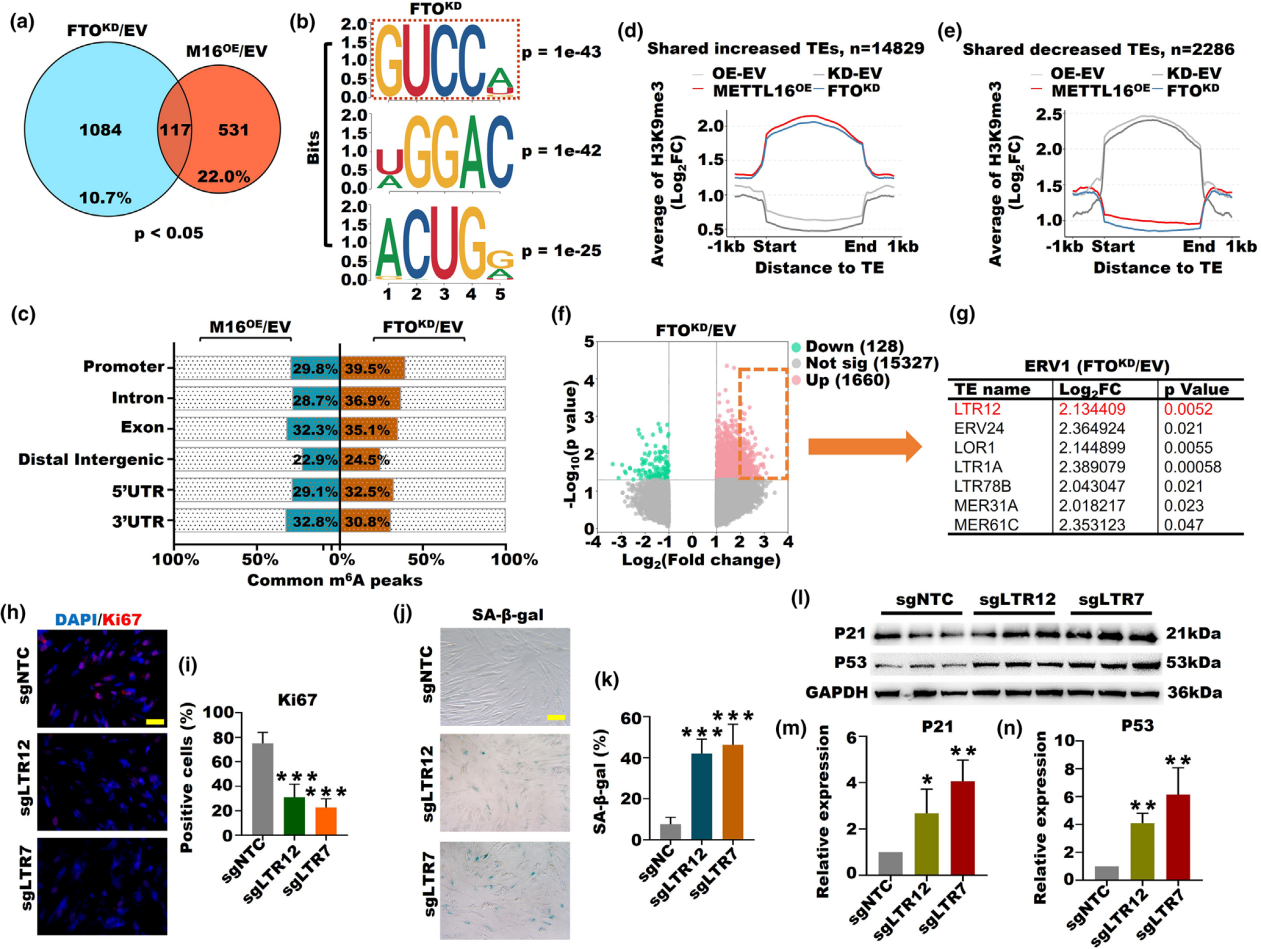
The activation of ERV1 induced by high m^6^A methylation levels is a trigger for ovarian aging. (a) Venn diagram analysis of the set of differentially expressed m^6^A‐modified genes (*p* < 0.05) within the FTO^KD^ and M16^OE^ groups. (b) The top three m^6^A motifs in the FTO^KD^ groups. (c) The proportion of shared m^6^A peaks across various genomic regions in the FTO^KD^ and M16^OE^ groups. (d) The average H3K9me3 signal on the shared increased TEs in the M16^OE^ and FTO^KD^ cell lines. (e) The average H3K9me3 signal on the shared decreased TEs in the M16^OE^ and FTO^KD^ cell lines. (f) Scatterplots showing differentially expressed TEs in the FTO^KD^ group. (g) The seven differentially expressed subfamilies of ERV1 in the FTO^KD^ group with FC >2. (h,i) Immunofluorescence of the proliferation marker KI67 in sgLTR12 and sgLTR7cells (***p < 0.001). (j,k) The staining results of the aging marker SA‐β‐gal in sgLTR12 and sgLTR7 cells (***p < 0.001). (l,m) Expression of the aging proteins p21 and p53 in sgLTR12 and sgLTR7 cells (*p < 0.05, **p < 0.01).

To explore potential differences in the mechanisms of m^6^A regulation between FTO^KD^ and M16^OE^, we conducted a preliminary Venn diagram analysis on the set of differentially expressed m^6^A‐modified genes (*p* < 0.05) within the FTO^KD^ and M16^OE^ groups. Our analysis revealed an overlap of merely 117 genes between these two groups, constituting approximately 10.7% of FTO^KD^ genes and 22% of M16^OE^ genes (Figure 7a). Additionally, we examined changes in m^6^A motifs within the FTO^KD^ and M16^OE^ groups. Interestingly, among the top three motifs in each group, only the GUCCA motif was common between them (Figure 7b, S13A). However, the proportion of shared m^6^A peaks between the FTO^KD^ and M16^OE^ groups across various genomic regions was approximately 30% (Figure 7c). Certain newly integrated HERV provirus subfamilies, such as the emerging subgroup called human endogenous retrovirus 1 (HERV1), maintain intact open reading frames that encode vital proteins necessary for viral particle assembly, notably gag, pol, and env (Vargiu et al., 2016). Interestingly, both the FTO^KD^ and M16^OE^ groups exhibited activated expression of HERV1‐pol, HERV1‐env, and HERV1‐gag (Figure S13b). Additionally, we observed that the mutation of FTO primarily restored the expression level of HERV1‐pol, while it had no impact on HERV1‐env and HERV1‐gag (Figure S13c). Next, we aimed to examine whether FTO^KD^ and M16^OE^ induce OA through common TEs. Our findings revealed that FTO^KD^ and M16^OE^ shared a total of 14,829 increased TEs, significantly surpassing the count of shared 2286 decreased TEs (Figure 7d,e). Subsequently, we observed a significant prevalence of increased TE counts in both the FTO^KD^ and M16^OE^ groups, each being approximately 13‐fold and 9‐fold higher than the count of downregulated counterparts, respectively (Figure 7f, S13D). Next, we selected ERV1 subfamily members from the upregulated group, focusing on those with notably substantial increases (Fold change >4 and *p* < 0.05). Interestingly, we observed a remarkable upregulation of LTR12 in both the FTO^KD^ and M16^OE^ groups, while LTR7 showed significant upregulation only in the M16^OE^ group compared to the EV (Figure 7g; S13e). Given previous evidence showing that LTR7 hindered self‐renewal and regeneration in hESCs (Sun et al., 2023), to elucidate the role of LTR7 and LTR12 in OA, we established KGN cell lines for activation of LTR7 and LTR12 according to previous research (Liu et al., 2023), denoted as sgLTR7 and sgLTR12, respectively. Furthermore, we observed a significant decrease in the proliferation marker KI67 in sgLTR12 and sgLTR7 cells (Figure 7h,i), accompanied by a notable increase in the cellular aging levels of both sgLTR12 and sgLTR7 (Figure 7j,k). Moreover, we noted a remarkable upregulation of aging‐associated proteins, including p21, p53, and p27, in both sgLTR12 and sgLTR7 cells (Figure 7l–n; Figure S13f,g). In addition, the LC3B showed a similar trend, displaying an upward trajectory in both sgLTR12 and sgLTR7 cells (Figure S13h,i). Therefore, the combination of FTO knockdown and METTL16 overexpression jointly enhanced m^6^A levels and subsequently decreased the expression of the H3K9me3 methyltransferase SUV39H1. This led to an reduction in H3K9me3 expression and activated the ERV1 family, particularly LTR7 and LTR12. Following the activation of LTR7 and LTR12, cell proliferation decreased, while the levels of apoptosis, cellular aging, and autophagy markers significantly increased. These mechanisms collectively contributed to the progression of OA.

## 3 DISCUSSION

To elucidate the role of m^6^A and its mechanisms in OD and aging, we conducted a comprehensive analysis of the m^6^A transcriptome across 10 human ovarian samples, spanning stages from fetal to Mop. Impressively, our investigations revealed an intriguing phenomenon: a substantial increase in m^6^A methylation levels was associated with OA (Figure 1c). Similarly, our previous research also identified that increased m^6^A modification resulting from FTO deficiency impeded spermatogenesis and Leydig cell maturation (Wu, Li, et al., 2023). Conversely, in the context of skeletal muscle in cynomolgus monkeys, METTL3 deficiency led to a reduction in m^6^A levels during aging, consequently contributing to cellular senescence (Wu, Lu, et al., 2023). Therefore, these evidence suggests that abnormal RNA m^6^A methylation levels can disrupt organismal homeostasis, ultimately leading to tissue aging, consistent with previous reports (Ma et al., 2018). Dynamic m^6^A modifications tend to maintain equilibrium within the organism and may vary across different mammalian tissues (Liu et al., 2020). Indeed, various m^6^A regulators, including FTO and METTL3, exhibit specificity toward different genes or substrates (Liu et al., 2014; Wei et al., 2018). Dysregulated expression of these m^6^A regulators could result in an excessive or insufficient m^6^A imbalance, both of which could contribute to the initiation of aging processes. Our study also confirmed that increased m^6^A modifications contribute to the onset of OA. These observations prompted us to contemplate the underlying mechanism through which heightened m^6^A modifications promote OA. Recently, an increasing amount of evidence has indicated that autophagic function diminishes with age in a wide range of organisms (Leidal et al., 2018). Autophagy has a substantial impact on the lifespan of various model organisms, with accelerating aging and its stimulation potentially exerting potent anti‐aging effects (Madeo et al., 2010). In addition to revealing that autophagy accelerated OA, we also substantiated a novel phenomenon: the heightened m^6^A methylation of RNA induced by M16^OE^ induced apoptosis and autophagy, which in turn led to OA (Figures S9 and S11). Previous research has proposed that YTHDF3 stimulates autophagy by recognizing m^6^A modification situated near the stop codon of FOXO3 mRNA (Hao et al., 2022), while YTHDC1 modulates autophagy by controlling the stability of SQSTM1/p62 nuclear mRNA (Liang et al., 2022). Remarkably, there have been no reports elucidating the relationship between METTL16 and autophagy. In conclusion, our study has uncovered novel insight into the association between METTL16‐induced m^6^A modification and autophagy as a driver of OA.

A dynamic epigenome is essential for development and health (Allis & Jenuwein, 2016), and investigating the interplay between RNA methylation and DNA epigenetic modifications is meaningful. In our study, we revealed that FTO^KD^ and M16^OE^ modulated the SUV39H1‐H3K9me3‐ERV1 axis in OA (Figures 5 and 6). H3K9me3 modifications, acting as epigenetic marks, are notably abundant at extensively repeated DNA sequences, playing a pivotal role in curtailing recombination and gene expression (Becker et al., 2016). Prior studies have highlighted the intricate relationship between the m^6^A and histone modifications. For instance, the m^6^A reader YTHDC1 physically interacted with and recruited KDM3B to m^6^A‐associated chromatin, facilitating H3K9me2 demethylation (Li et al., 2020). In mouse embryonic stem cells, METTL3 recruited the SETDB1/TRIM28 complex to govern H3K9me3 modification, effectively silencing IAPEz, a subfamily of non‐LTR retrotransposons (Xu et al., 2021). In our study, we made a novel discovery by observing a decrease in SUV39H1 expression induced by high m^6^A methylation in both the FTO^KD^ and M16^OE^ groups (Figures S9 and S12), which subsequently resulted in reduced H3K9me3 levels in both (Figures 5 and 6). Significantly, a notable reduction in global H3K9me3 levels has been identified in conditions linked to premature aging (Zhang et al., 2015), providing strong support for our point. However, the regulators of H3K9me3, specifically SUV39H1, in the context of OA differed from the reports mentioned above. One plausible explanation for this disparity may be the differences in the regulation and recruitment pathways of H3K9me3 in distinct physiological states, such as aging and development.

As the levels of H3K9me3 methylation decrease, their ability to bind to heterochromatin DNA is weakened, which could potentially result in the activation of TE (Ninova et al., 2019). Consistent with these reported findings, we found that both FTO^KD^ and M16^OE^ activated the ERV1 family (Figure S13) and shared a significant overlap in the majority of increased TE (Figure 7). Prior research has suggested that the reactivation of ERVs can drive cellular, tissue, and organismal aging (De Cecco et al., 2013; Liu et al., 2023). Nevertheless, the mechanism through which m^6^A modification of ERV1 influences the process of OA remains unexplored. Simultaneously, our discoveries revealed that the activation of ERV1 was crucial in OA, and this regulatory mechanism was influenced by FTO^KD^ and M16^OE^. Recently, a report indicated that the accumulation of HERVK was associated with cellular and tissue aging (Liu et al., 2023). However, despite ERV1 and HERVK belonging to LTR retrotransposons, they exhibited different abundance ratios in various species and have integrated into the genome at distinct stages of biological evolution (Mouse Genome Sequencing et al., 2002). Furthermore, various retrotransposon subfamilies, including both LTR and non‐LTR elements such as LINE1, showed varying expression patterns depending on specific developmental stages and cell types, controlled by different m^6^A regulators (Goke et al., 2015; Wei et al., 2022). Thus, we speculated that certain ERV subfamilies played a crucial role in OA. A growing body of evidence suggests that NANOG triggers the activation of LTR7 in primed hESCs and potentially in postimplantation blastocysts (Ai et al., 2022), while LTR7 activation exerts an inhibitory effect on hESC self‐renewal and regeneration (Sun et al., 2023). With the collective findings and considering the connection between the loss of pluripotency and aging (Lo Sardo et al., 2017), we surmised that the activation of LTR7 could also be a pivotal factor in OA. Indeed, for the first time, we revealed that LTR7, a component of the ERV1 family, demonstrated passive activation in both the FTO^KD^ and M16^OE^ groups, accompanied by an upregulation of m^6^A methylation (Figure 7; S13). A hallmark of mammalian aging is the loss of cellular pluripotency and the reduced capacity for regeneration (Studer et al., 2015), and the inhibition of hESC regeneration and the acceleration of OA by LTR7 activation further solidified this concept. Importantly, we observed that the mechanisms leading to LTR7 activation differed between developmental arrest and accelerated aging. This disparity in the regulatory mechanisms of aging and regeneration may be attributed to diametrically opposite physiological stages within the organism. In summary, our discovery of the ERV1 family's role associated with m^6^A in OA has opened a new avenue of research: FTO^KD^ and M16^OE^ collaboratively increased m^6^A levels, downregulated SUV39H1, and subsequently decreased H3K9me3 levels. This cascade initiated the activation of the ERV1 family, specifically LTR7, resulting in reduced cell proliferation and heightened indicators of apoptosis, cellular aging, and autophagy (Figure abstract).

### 3.1 Limitations of the study

FTO, as an RNA demethylase, facilitates the removal of methyl groups such as N6‐methyladenosine (m^6^A), N6‐2‐O‐dimethyladenosine (m^6^Am) and N1‐methyladenosine (m^1^A) (Wei et al., 2018). In our study, we focused solely on investigating the mechanism by which FTO regulates OA through m^6^A modifications. Future research can delve deeper into exploring how FTO affects m^6^Am and m^1^A modifications to elucidate the multifaceted aspects of this physiological process.

## 4 METHODS

### 4.1 Ethics statement

This research received approval from the Ethical Committee of Suzhou Municipal Hospital (approval no. 2016004). Informed consent was obtained in accordance with the principles of the Declaration of Helsinki.

### 4.2 Collecting ovarian tissue from fetal and adult donors

Seven human ovary samples were excised from patients who underwent procedures unrelated to ovarian conditions and provided informed consent. These samples included fetuses with 100% primordial follicles (*n* = 3, second trimester), young women with a notable increase in preantral follicles, antral follicles22‐30, and corpus luteum (*n* = 3, 22–30 years old), older women with an increase in corpus albicans (*n* = 2, 31–40 years old), and Mop women with predominantly corpus albicans (*n* = 2, 50–60 years old). Human adult ovary samples were obtained from patients who underwent bilateral oophorectomy and provided informed consent. These samples were from normal ovaries without pathological changes. Patients with acute infectious diseases, malignant tumors, hereditary diseases, and systemic immune diseases were excluded from the study. Additionally, patients with conditions such as polycystic ovary syndrome, premature ovarian failure, endometriosis, and other reproductive endocrine disorders, including thyroid disease, diabetes, adrenal disease, and so on, were also excluded.

### 4.3 FTO^KO^ mice

The generation of FTO knockout (FTO^KO^) mice has been described in previous reports (Wei et al., 2022). In summary, FTO^−/−^ mice were obtained through crossing FTO heterozygous mice. These mice were housed under specific pathogen‐free (SPF) conditions, with controlled environmental parameters such as a temperature range of 20–22°C, a 12‐h light/12‐h dark cycle, humidity levels maintained between 50%–70%, and unrestricted access to food and water. Genomic DNA was extracted from the mice's toes for genotyping.

### 4.4 Construction of FTO^KD^ , FTO^OE^ , FTO^MUT^ , and METTL16^OE^ plasmids

To generate human FTO^KD^ vectors, specific short hairpin RNAs (shRNAs) were inserted into the AgeI/EcoRI sites of the lentiviral vector pLKO.1. For the construction of the FTO^OE^ and FTO^MUT^ plasmids, we followed a procedure outlined in previous literature (Jia et al., 2011). In brief, the FTO fragment was amplified from human genomic DNA using the FTO‐Fwd and FTO‐Rev primers. The amplified FTO fragment was subsequently integrated into the mammalian vector pcDNA3 (Beijing Tsingke Biotech). To create the FTO^MUT^ plasmid, specific mutations, H231A and D233A, were introduced into the plasmid. METTL16 was amplified from human genomic DNA, guided by GenBank Accession No. NP_076991.3. The amplified METTL16 fragment was then inserted into the mammalian vector pEnCMV (MiaoLing Biology) to generate METTL16^OE^ plasmids. The primers and plasmids utilized in this study are documented in Table S4.

### 4.5 Construction of sgLTR7 and sgLTR12 pLasmids

The sgLTR7 and sgLTR12 plasmids were constructed following the procedures described in previous literature (Liu et al., 2023). To activate endogenous HERV1, we cloned nontargeting control sgRNA (sgNTC) or sgRNA targeting HERV1 LTR7 or LTR12F (sgHERV1) into the LentiSAM v2 vector (Addgene, #75112) through the ESP3I site. Subsequently, these constructs were cotransfected with LentiMPH v2 (Addgene, #89308) in KGN cells. We assessed the suitability of the sgRNAs for the HERV1 CRISPRa system by initially obtaining the genomic coordinates of RepeatMasker‐annotated repetitive elements using the hg19 assembly. Next, we constructed the repetitive elements of LTR7 and LTR12F (The promoters of HERV1) using the BLAST (Basic Local Alignment Search Tool) database. Table S4 provides the sequences of the sgRNAs utilized for CRISPRa and the primers employed for plasmid construction.

### 4.6 Cell culture and plasmid transfection

The KGN cell line, a human ovarian granulosa tumor cell line, was procured from Suzhou Yimer Technology (Suzhou, China). KGN cells were cultured in DMEM (Gibco, 11,995,065) supplemented with 10% (v/v) FBS (Gibco, 10,099,141) at 37°C in a 5% CO_2_ environment. For transfection, KGN cells were seeded in 6‐well cell culture plates (Corning, 3516) 16–20 h before transfection at a cell density of approximately 80%. The transfection process was facilitated using Lipofectamin 2000 Transfection Reagent (Invitrogen, 11,668,019). For every 1 μg of plasmid, 3 μL of transfection reagent was added. After 24 h of incubation with the plasmid‐liposome mixture, the culture medium was replaced with fresh medium, and the cells were collected after 72 h. Details regarding the plasmids utilized can be found in Table S4.

### 4.7 RNA isolation and DNase digestion

Total RNA was extracted from ovarian tissue and the KGN cell line using TRIzol reagent (Invitrogen, 15,596,018) following the manufacturer's instructions. Samples with high quality (28S/18S >2) were chosen for subsequent experiments. To ensure the removal of DNA contamination, Turbo DNase treatment (Invitrogen, AM2239) was carried out. The concentration of RNA was quantified using the Qubit RNA HS Assay Kit (Thermo Fisher Scientific, Q32855).

### 4.8 m^6^A‐RIP‐seq

The procedure of MeRIP sequencing was slightly modified from the previously described low‐input m^6^A‐seq protocol (Zeng et al., 2018). Briefly, 12.5 μg of total RNA was fragmented into 200 nt pieces by RNA Fragmentation Reagents (Thermo Fisher Scientific, AM8740). The fragmentation reaction was carried out for approximately 5–6 min in a preheated thermal cycler (Eastwin, ETC 821) at 70°C. Then, 2 μL of Stop Solution (0.5 M EDTA) was added to stop the reaction. The fragmented RNA was pelleted by ethanol precipitation. For m^6^A input, 10 ng fragmented RNA was used to construct the strand‐specific RNA library with SMARTer Stranded Total RNA‐Seq Kit v2‐Pico Input Mammalian (Takara‐Clontech, 634,488). The remaining RNA was employed for m^6^A‐seq: 30 μL of protein G magnetic beads (Thermo Fisher Scientific, 10004D) and 30 μL of protein A magnetic beads (Thermo Fisher Scientific, 10002D) were mixed and washed twice with IP buffer (10 mM pH 7.5 Tris–HCl, 150 mM NaCl, and 0.1% IGEPAL CA‐630), and then the mixed beads were resuspended in 500 μL of IP buffer. Five micrograms of anti‐m^6^A antibody (Millipore, MABE1006) was added to the resuspended beads and rotated at 4°C for approximately 6 h. After the combination of beads and antibody, the bead‐antibody mixtures were washed twice with IP buffer and resuspended in 500 μL of IP reaction buffer containing fragmented RNA and 5 μL of recombinant RNase inhibitor (Takara‐Clontech, 2313B). After rotating at 4°C overnight, the bead‐antibody‐RNA mixtures were washed twice with IP buffer, washed twice with low‐salt IP buffer (10 mM pH 7.5 Tris–HCl, 50 mM NaCl and 0.1% IGEPAL CA‐630), and washed twice with high‐salt IP buffer (10 mM pH 7.5 Tris–HCl, 500 mM NaCl and 0.1% IGEPAL CA‐630). After low/high salt washing, the bound RNA was eluted by competition with 6.7 mM N^6^‐methyladenosine (Selleckchem, S3190). The 200 μL eluted RNA was mixed thoroughly with 1400 μL 100% ethanol and 700 μL RLT buffer. The mixture was loaded onto an RNeasy MiniElute spin column (QIAGEN, 74104) and centrifuged at 4°C–12,000 rpm for 1 min. Afterward, the membrane of the spin column was washed with 500 μL of RPE buffer one time, then 500 μL of 80% ethanol once, and finally centrifuged at 4°C at full speed for 5 min. Fourteen microliters of ultrapure H_2_O was used to elute m^6^A RNA. The input RNA (10 ng fragmented RNA) and m^6^A RNA were used as starting materials to construct libraries with SMARTer Stranded Total RNA‐Seq Kit v2‐Pico Input Mammalian (Takara‐Clontech, 634,488) according to the standard protocol. The number of PCR cycles for input RNA was 11, whereas that for m^6^A RNA was 14. The libraries were sequenced on an Illumina NovaSeq with a PE 150 bp read length.

### 4.9 m^6^A dot blot

The procedure for total RNA analysis was as follows: Total RNA was first diluted to a uniform concentration using ultrapure H_2_O (Beyotime, ST876). Next, the diluted RNA was denatured at 95°C for 3 min. Subsequently, 2 μL of total RNA was applied to Hybond N+ membranes (GE Healthcare, RPN303C). The nitrocellulose membranes underwent two rounds of cross‐linking in a 2400 UV crosslinker. The membranes were then blocked with 5% skim milk (BD Difco, 232,100) and incubated overnight with a primary anti‐m6A antibody (Cell Signaling Technology, 56593S) at a 1:3000 dilution. Following three 10‐minute washes in PBST, the membranes were exposed to the corresponding HRP‐labeled secondary antibody at room temperature for 1 h. To normalize the results, methylene blue staining in PBS for 10 min was performed, followed by a rinse in ribonuclease‐free water for 5 min. Finally, the blots were captured using an exposure system and analyzed using ImageJ. The blotting procedure was carried out in the same manner.

### 4.10 RT‐qPCR

Total RNA was extracted from tissues or cells using RNAiso plus (Takara‐Clontech, 9109). First‐strand complementary DNA was synthesized using the PrimeScrip First Strand DNA Synthesis Kit (Takara‐Clontech, 6210A). Quantitative reverse transcription (qRT) was conducted following the manufacturer's instructions, employing SYBR premix (BioSharp, BL698A). Polymerase chain reaction (qPCR) was performed using the primer sequences listed in Table S5.

### 4.11 HE staining of mouse ovaries

Ovaries from female mice aged 6 and 12 months were collected. One side of the ovaries was preserved in liquid nitrogen, while the other side was immediately fixed in 4% paraformaldehyde (Sangon Biotech, E672002‐0500). After an overnight incubation at 4°C, the fixed ovaries were rinsed with tap water for 4 h. Subsequently, the ovaries were subjected to a dehydration process using a gradient of ethanol, followed by hyalinization with xylene, and finally embedded in paraffin. The paraffin‐embedded tissues were then sectioned into 5 μm thick slices, deparaffinized, rehydrated, subjected to HE staining (Beyotime, C0105S), and visualized using a microscope (Olympus, Japan).

### 4.12 Oocyte collection and processing

Germinal vesicle (GV) oocytes from the ovaries of 6‐week‐old female mice were obtained 48 h after intraperitoneal injection of pregnant mare serum gonadotropin (PMSG) (Nanjing Aibei, M2620). These oocytes were then transferred to M2 culture medium (Sigma‐Aldrich, M7167). Intermediate II (MII) oocytes were collected after 14–16 h and incubated in M2 culture medium at 37°C for 3–5 min in preparation for subsequent experiments.

### 4.13 Immunofluorescence staining of mouse ovaries and oocytes

Paraffin‐embedded mouse ovary sections were pretreated and then incubated with the primary antibody overnight at 4°C. This was followed by incubation with the secondary antibody for 1 h at room temperature. Sections were blocked with a blocking agent containing Hoechst 33342 staining solution (Beyotime, C1025) and were visualized under a microscope. Photographs were captured using an Olympus digital camera. Details of the primary antibodies used can be found in Table S6. For oocytes, including immature (IF and GV) and mature (MII) oocytes, the procedure involved fixation in 4% paraformaldehyde, followed by five washes in PBS. The oocyte membranes were permeabilized with 0.5% Triton X‐100 (Sigma‐Aldrich, 93,443) for 30 min at room temperature. Oocytes were then incubated with primary antibodies overnight at 4°C and subsequently with corresponding secondary antibodies for 2 h at room temperature. Confocal imaging of oocytes was performed using a ZEISS LSM 800 microscope.

### 4.14 Electron microscopy

The mouse ovaries were washed with saline. They were then gently cut into small tissue blocks, each less than 1 mm^3^ in size, using a razor blade. These small tissue blocks were placed in a preprepared fixative containing 1 mol/L phosphate‐buffered glutaraldehyde and were fixed at 4°C for over 2 h. After fixation, the tissue blocks were removed from the 4°C refrigerator and rinsed three times with phosphate buffer solution, with each rinse lasting approximately 15 min. The tissue blocks were then subjected to a gradient dehydration process using ethanol, starting from 30% ethanol and progressing to 100% ethanol. Each ethanol concentration was used for 15–20 min, with a final step of 100% ethanol for an additional 15–20 min. Subsequently, the samples were placed in a 1:1 volume ratio mixture of 1% ethanol and 100% acetone for 15–20 min. After dehydration, the samples were permeated with resin and placed in a 2:1 ratio of 100% acetone to Epon812 resin at room temperature for 3–4 h. Following this, the samples were treated with a 1:2 ratio of 100% acetone to Epon812 resin at room temperature for 3–4 h. The samples were then immersed in pure embedding agent (Epon812 resin) twice, each time for 3–4 h. Finally, the tissue blocks were embedded in an embedding mold. The oven was preheated to the desired temperature, and the samples were first fixed at 37°C for 12 h, transferred to 45°C for 12 h, and finally fixed at 60°C for 48 h. After embedding and sectioning, the samples were stained with 2% uranyl hydrogen peroxide hydrochloride for 20 min, followed by lead citrate staining for 15 min. The prepared slices were observed using a transmission electron microscope, specifically the HT7700 model from HITACHI in Tokyo, Japan.

### 4.15 Nascent RNA labeling assay

KGN cells were cultured in six‐well plates equipped with cell crawlers. After twenty‐four hours of plasmid transfection, de novo RNA synthesis assays were conducted using the EU nascent RNA Detection kit (RIBOBIO, C10316‐1 or C10316‐3) following the manufacturer's instructions. Images were captured using a microscope (Olympus, Japan), and the image intensities were quantified using ImageJ software.

### 4.16 Western blot

Cell and tissue samples were lysed using RIPA buffer (composition: 50 mM Tris–HCl pH 7.4, 150 mM NaCl, 0.25% deoxycholic acid, 1% NP‐40, 1 mM EDTA) (Beyotime, P0013C) supplemented with 1× protease inhibitor (Roche, 11,873,580,001). Equal amounts of lysate were mixed with sample buffer (comprising 0.05% bromophenol blue, 2% sodium dodecyl sulfate, 50 mM Tris–HCl pH 6.8, 5% β‐mercaptoethanol) and heated to 100°C for 10 min. Subsequently, the samples were separated on a 12% sodium dodecyl sulfate‐polyacrylamide gel electrophoresis (SDS‐PAGE) gel and transferred to a PVDF membrane (Millipore, IPVH00010) using rapid transfer buffer (NeoSeamy, WB4600). The PVDF membrane was incubated with TBST (Tris‐buffered saline, 0.1% Tween 20, 5% skim milk) for 1 h at room temperature. Primary and secondary antibodies (refer to Table S6 for details) were used for antibody detection. Protein signals in each sample were visualized using a SuperFemto ECL Chemiluminescence Kit (Vazyme, E423), and the images were captured with a chemiluminescent detection system. This experiment was repeated three times, and the results are presented as the fold change ± SD.

### 4.17 Senescence‐associated β‐galactosidase (SA‐β‐gal) assay

KGN cells were stained 72 h following plasmid transfection using the SA‐β‐Gal staining kit (Beyotime, C0602) following the manufacturer's guidelines. The procedure involved the following steps: Cells were first washed three times with PBS. Subsequently, they were fixed using SA‐β‐Gal fixative for 15 min at room temperature. After fixation, cells underwent another three rounds of washing with PBS, each lasting for 3 min. Next, the cells were incubated in SA‐β‐Gal working solution at 37°C overnight. The resulting images were captured using a microscope (Olympus, Japan), and ImageJ software was used for subsequent analysis.

### 4.18 CUT and tag assays

After 72 h of KGN cell plasmid transfection, approximately 1 × 10^5^ cells were extracted from each sample set. These cells were then treated with 10 μL of concanavalin A‐coated magnetic beads (provided by Vazyme, TD903) for 10 min, following the manufacturer's guidelines. Subsequently, the cells were incubated with a digoxigenin buffer containing the following components: 20 mM HEPES (pH 7.5), 150 mM NaCl, 0.5 mM spermidine, 1× enzyme inhibitor cocktail, 0.05% digitalisin, and 2 mM EDTA. The cells were then subjected to an overnight incubation at 4°C with a 1:50 dilution of either anti‐H3K9me3 or IgG antibody suspension beads. After the primary antibody incubation, the cells had their primary antibodies removed using a magnet, followed by incubation with a secondary antibody (diluted 1:100) for 60 min. Next, the cells were incubated with the pA‐Tn5 complex for 1 h. Afterward, the cells were washed using DIG‐MED buffer and resuspended in a labeling buffer consisting of 10 mM MgCl_2_ within DIG‐MED buffer. The cells were then incubated at 37°C for 1 h. Genomic DNA was extracted through phenol‐chloroform‐isoamyl alcohol extraction, followed by ethanol precipitation. Sequencing libraries were prepared in accordance with the manufacturer's instructions and purified using XP beads from Beckman Counter. Finally, sequencing was carried out on an Illumina NovaSeq 6000 using PE150 mode.

### 4.19 Read preprocessing and alignment

Initially, all the raw data reads from both the input and m^6^A‐seq datasets were mapped to the human reference genome (GRCh37.75, Ensembl) using Bowtie2 (Version 2.4.1). Any reads that aligned to rRNA were subsequently removed from the analysis. The remaining reads were then aligned to the human genome using HISAT2 (Version 2.2.1) with default parameters. The results obtained from HISAT2 were subsequently converted into BAM format using BEDTools (Version 2.29.2).

### 4.20 The process of identifying m^6^A peaks in mRNAs

Initially, the raw sequencing reads underwent cleaning of the UMI barcode using fastq (Version 0.12.4) with the parameters “‐Q‐U‐umi_loc=read2‐umi_len=8‐umi_prefix=UMI.” The reads were then initially processed through Trim_galore (http://www.bioinformatics.babraham.ac.uk/projects/trim_galore/). The quality threshold was set at 20, and post‐trimming, the reads were required to be at least 30 nt in length. Subsequently, the output reads were trimmed using Trimmomatic (version 0.36) to eliminate low‐quality bases and adapters with the parameters “LEADING:20 TRAILING:20 SLIDINGWINDOW:4:20 MINLEN:30.” For the alignment of all m^6^A‐seq and input raw data reads against rRNA (hg38, obtained from UCSC Genome Browser), Bowtie2 (Version 2.4.1) was employed, while retaining the unmapped reads for further analysis. The remaining reads were aligned to the hg19 human genome using STAR (Version 2.7.8) with default parameters. Subsequently, the output from STAR was converted to BAM format using BEDTools (Version 2.29.2). To validate the putative m^6^A sites, the m^6^A‐enriched regions in each sample were identified using MACS2 (Version 2.2.7) with the corresponding control input sample. The effective size of the human genome was defined as 2.73 × 10 (Navarro‐Pando et al., 2021), with the option‐nomodel selected and a q‐value cutoff of 0.01. The m^6^A‐modified genes were annotated using the R package ChIPseeker, and the methylation level of each peak for each gene was calculated based on specific criteria. GO enrichment analysis was performed using the R packages clusterProfiler and ggplot2 to visualize the data. The distribution of m^6^A sites within mRNAs was assessed using the R package Guitar. Various visualization tools, including Pearson correlation heatmaps and violin plots, were employed to display the characteristics of m^6^A‐modified genes. Read coverage of m^6^A peaks was visualized using IGV software (Version 2.4.15).

### 4.21 The process of identifying m^6^A peaks in repeat RNAs

To identify m^6^A peaks in repeat RNAs, the clean reads were mapped to the UCSC human hg19 rmsk genome using STAR (Version 2.7.8a) with default settings. The results obtained from STAR were then converted to BAM format using BEDTools (Version 2.29.2). Subsequently, m^6^A in repeat RNA peaks was called using MACS2 (Version 2.2.7) with the ‘—nomodel’ parameter. Significant peaks with q‐values <0.01, as identified by MACS2, were considered for further analysis. The m^6^A methylation levels of these repeat RNAs were normalized and calculated following the method used for identifying m6A peaks in mRNAs (Described previously). To quantify the expression level for each locus, the number counts of repeat reads within the m^6^A peaks were calculated using featureCounts (Version 2.0.1) with the parameters “‐p‐t‐exon‐g gene_id.” For downstream analysis, the transcripts per million (TPM) value was calculated as the signal density in the sequencing data.

### 4.22 Determination of the m^6^A motif and its distribution pattern

Only m^6^A peaks that were consistently identified in a minimum of 3 biological replicates were considered for analysis. To identify the common peaks across different replicates, we calculated their intersection with the ‘‐f 0.5’ parameter and merged them using BEDTools (v.2.29.2). Subsequently, we utilized BEDTools to isolate the peaks that exhibited increased m^6^A modification relative to the WT group, employing the ‘‐A‐F 0.5’ parameter. These increased peaks were selected for further analysis. For the identification of de novo and known motifs associated with these increased peaks, we employed the findMotifsGenome.pl. script from Homer software (http://homer.ucsd.edu/homer/motif). During this motif search, we utilized the ‘hg19‐rna‐len 5’ parameters to ensure a comprehensive exploration of motif patterns within the genomic sequences.

### 4.23 Statistical analysis

For comparisons between two groups, a *t*‐test was used. In instances with three or more groups, a one‐way ANOVA was performed, followed by the t test for further analysis. All statistical analyses were carried out using GraphPad Prism software, and a significance level of *p* < 0.05 was considered to indicate a statistically significant difference.

## AUTHOR CONTRIBUTIONS

B.X.H. conceived and designed the experiments, analyzed the data, X.J.H., J.F.L., and C.D. conducted the majority of the experiments, B.X.H. and J.F.L. wrote the paper, J.C.L. and P.H. provided technical support for the analysis of MeRIP‐seq and Cut&tag seq data, H.L., Q.Y.Z., and C.F.Q. assisted with sample collection.

## CONFLICT OF INTEREST STATEMENT

The authors declare no competing interests.

## Supporting information


Data S1.

## Data Availability

The raw sequence data presented in this paper have been archived in the Genome Sequence Archive (Members & Partners, 2022) at the China National Center for Bioinformation / Beijing Institute of Genomics, Chinese Academy of Sciences, under accession number GSA‐Human: HRA005579. These data are publicly accessible at https://ngdc.cncb.ac.cn/gsa‐human.
