# Supplementary material for: The N6‐methyladenosine landscape of ovarian development and aging highlights the regulation by RNA stability and chromatin state
Source: Aging Cell. 2024 Oct 15;24(2):e14376. doi: 10.1111/acel.14376 (PMC11822672; doi:10.1111/acel.14376)
Supplement: Supplementary file 1 — Data S1. [file ACEL-24-e14376-s001.docx]

Supplementary Materials for

The N6-methyladenosine landscape of ovarian development and aging highlights the regulation by RNA stability and chromatin state

Xiujuan Hu *et al.*

*Corresponding author. Email: [huangboxiannj@163.com](mailto:huangboxiannj@163.com)

**This file includes:**

Supplementary Text

Figs. S1 to S13

Tables S1 to S6

References (1 to 7)

**Other Supplementary Materials for this manuscript include the following:**

Data S1

Software S1

Abbreviation

Fig. S1.


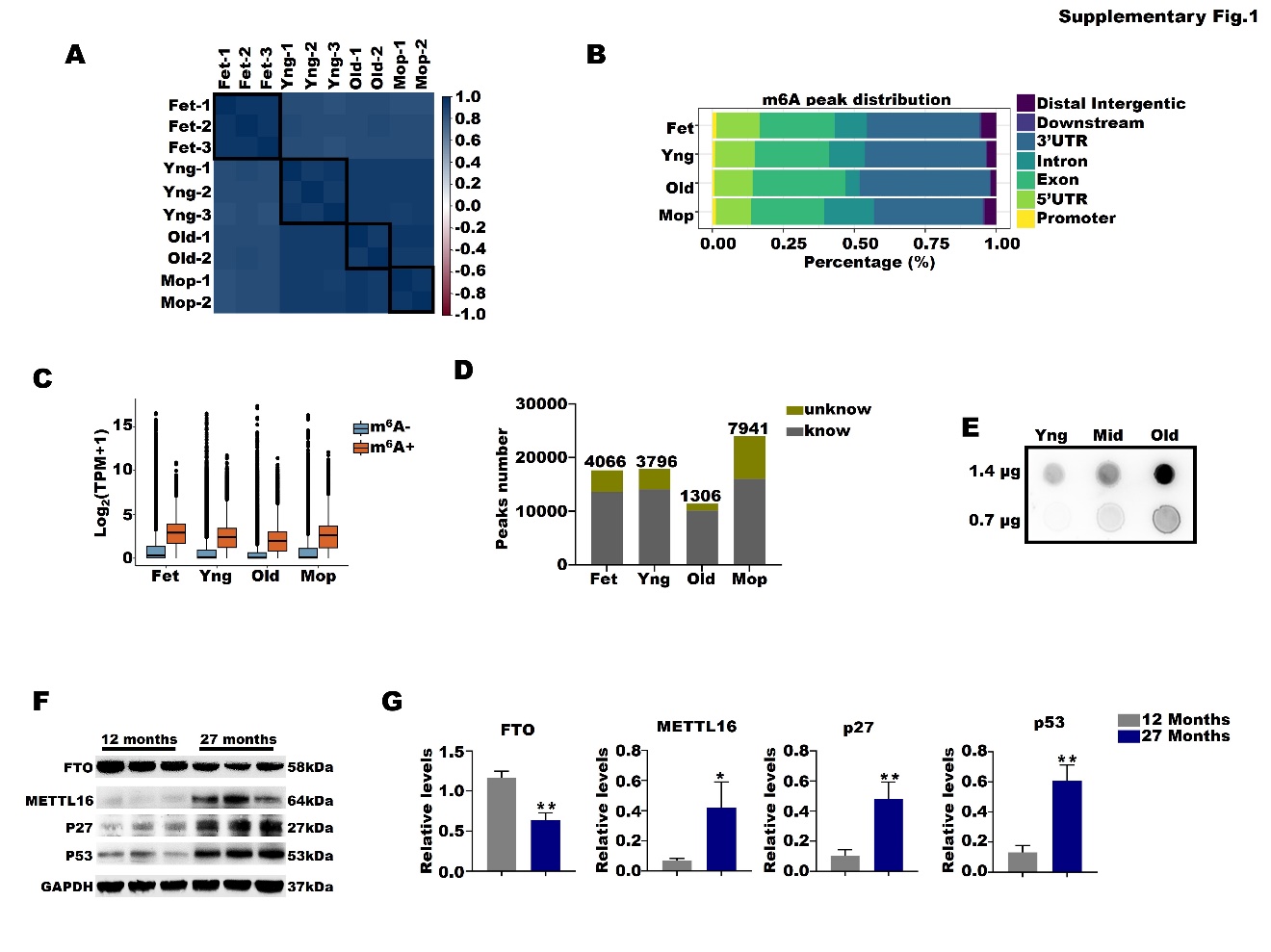


**Fig. S1. Transcriptome-wide profiling of mRNA levels in human ovaries at different stages and the m^6^A dot blot and western blot for mouse ovaries, related to Figure 1.**

A, Heatmap of Pearson correlation of the mRNA levels of the matched samples between human ovaries.

B, The distribution pattern of m^6^A peaks in each ovary across different genomic regions, including the promoter, intron, distal intergenic, 3′UTR, 5′UTR, CDS, and downstream regions.

C, Box plot showing the mRNA expression levels of m^6^A+ and m^6^A- genes in the Fet, Yng, Old, and Mop groups.

D, Known and unknown m^6^A peaks in the Fet, Yng, Old, and Mop groups mapped with the m6A-Atlas database.

E, The m^6^A dot blot in Yng, Mid and Old mouse ovaries.

F-G, Protein expression levels of FTO, METTL16, p27 and p53 in mouse ovaries at 12 and 27 months of age.


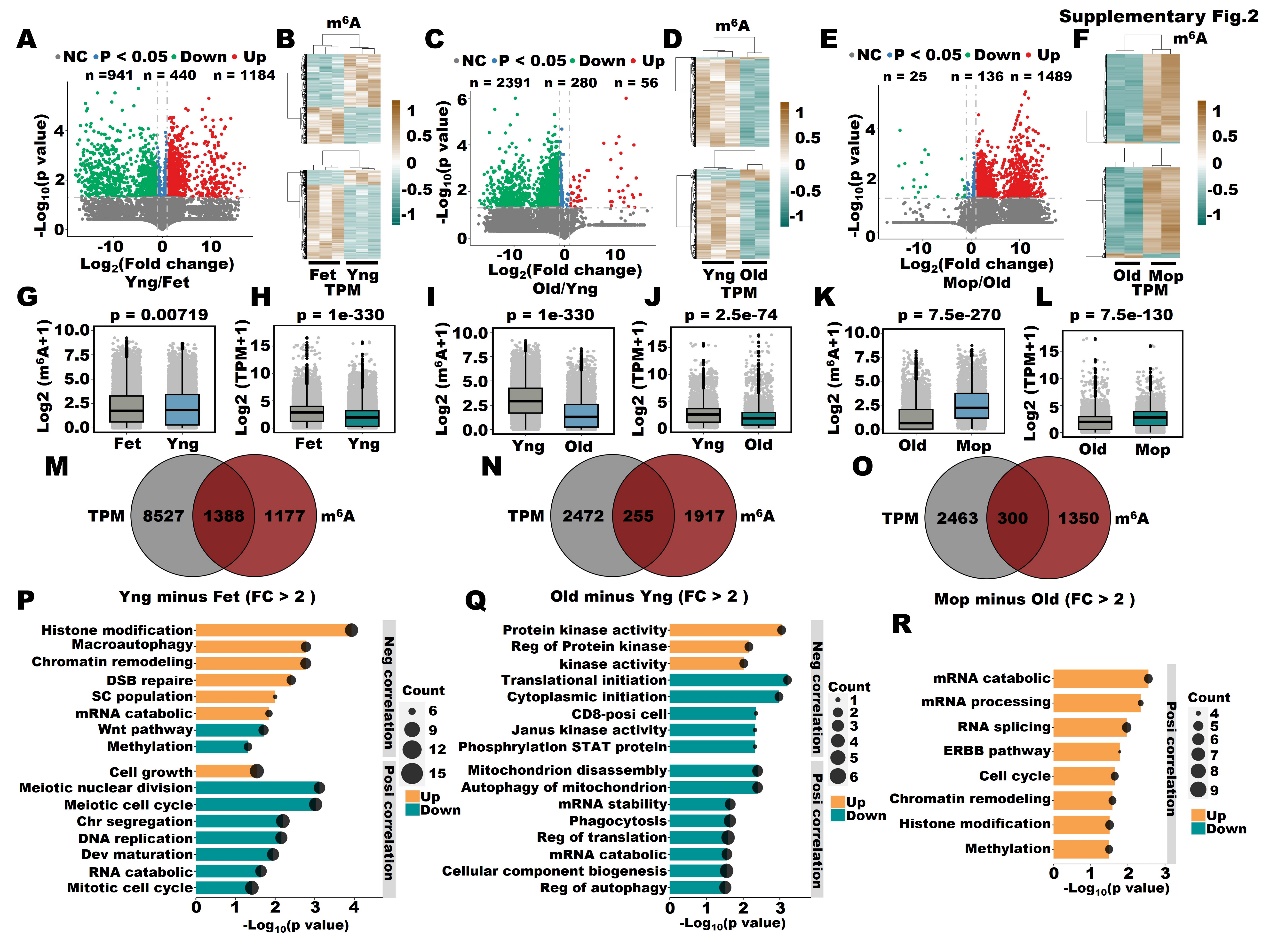


**Fig. S2. Coefficient correlation analysis of m^6^A and mRNA levels during OD, OFD, and OA and along with their associated pathways, related to Figure 1.**

A, Scatterplots showing upregulated and downregulated genes during the OD stage (*p* < 0.05).

B, Heatmap showing common genes with mRNA and m^6^A levels that are either upregulated or downregulated during the OD stage.

C, Scatterplots showing upregulated and downregulated genes during the OFD stage (*p* < 0.05).

D, Heatmap showing common genes with mRNA and m^6^A levels that are either upregulated or downregulated during the OFD stage.

E, Scatterplots showing upregulated and downregulated genes during the OA stage (*p* < 0.05).

F, Heatmap showing common genes with mRNA and m^6^A levels that are either upregulated or downregulated during the OA stage.

G-L, Box plot showing the expression levels of common genes with mRNA and m^6^A during OD, OFD, and OA.

M-O, Venn diagram of the differentially expressed m^6^A and mRNA levels between adjacent stages (fold change > 2, next stage minus previous stage).

P-R, GO analysis of overlapping genes during OD, OFD, and OA related to M-O.


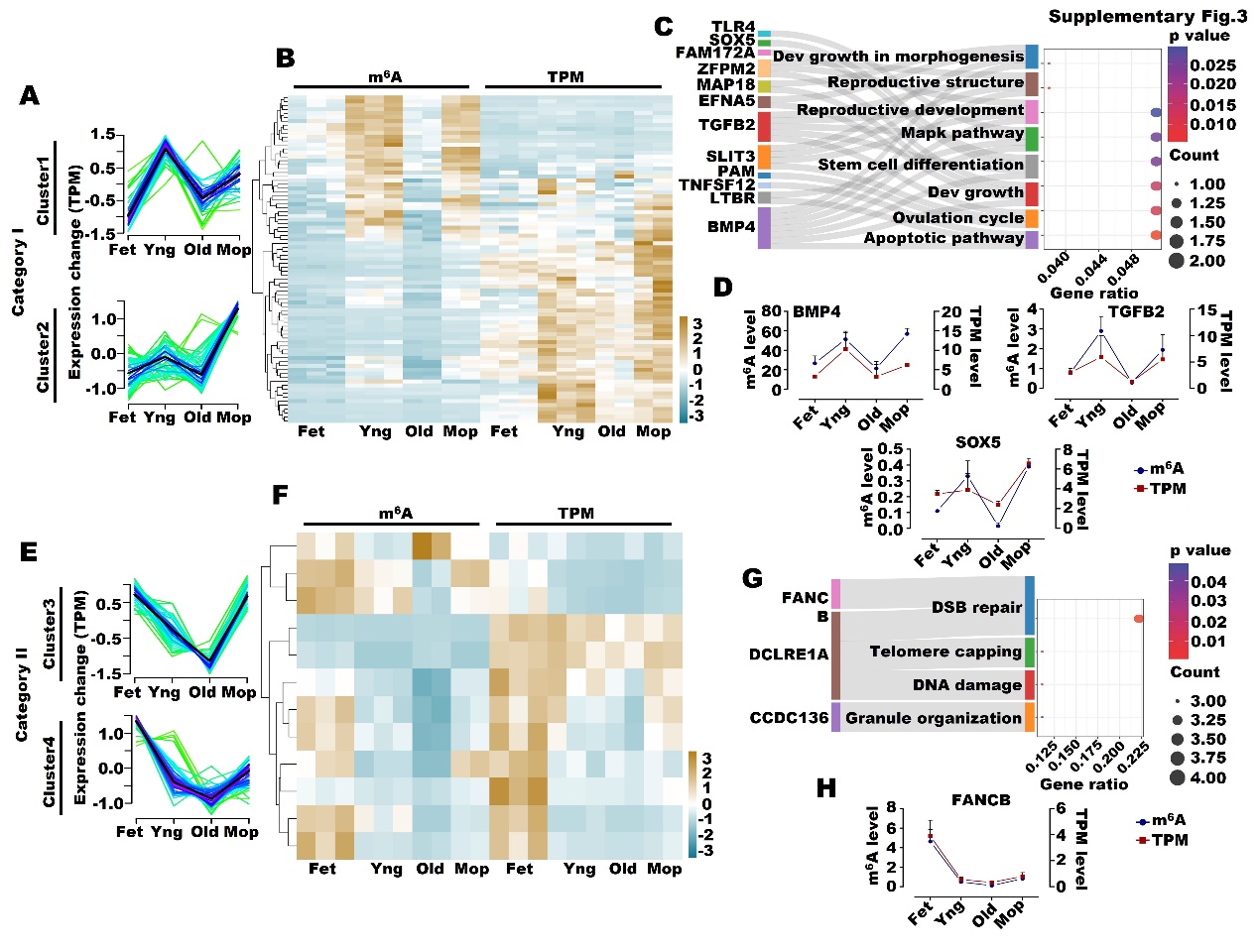


**Fig. S3. Fuzzy c-means clustering, heatmap and Gene Ontology enrichment analysis of the genes commonly expressed m^6^A and mRNA during OD, OFD, and OA, related to Figure 1.**

A, Fuzzy c-means clustering identified two temporal patterns of mRNA (category I). The x-axis represents four stages, while the y-axis represents log2-transformed, normalized intensity ratios in each stage.

B, The cluster heatmap of m^6^A levels and mRNA levels in category I.

C, The regulatory pathways of m^6^A modification in category I.

D, The m^6^A and mRNA levels of BPM4, TGFB2, and SOX5 during OD, OFD, and OA

E, Fuzzy c-means clustering identified two temporal patterns of mRNA (category II). The x-axis represents four stages, while the y-axis represents log_2_-transformed, normalized intensity ratios in each stage.

F, Cluster heatmap of m^6^A levels and mRNA levels in category II.

G, The regulatory pathways of m^6^A modification in category II.

H, The m^6^A and mRNA levels of FANCB during OD, OFD, and OA.


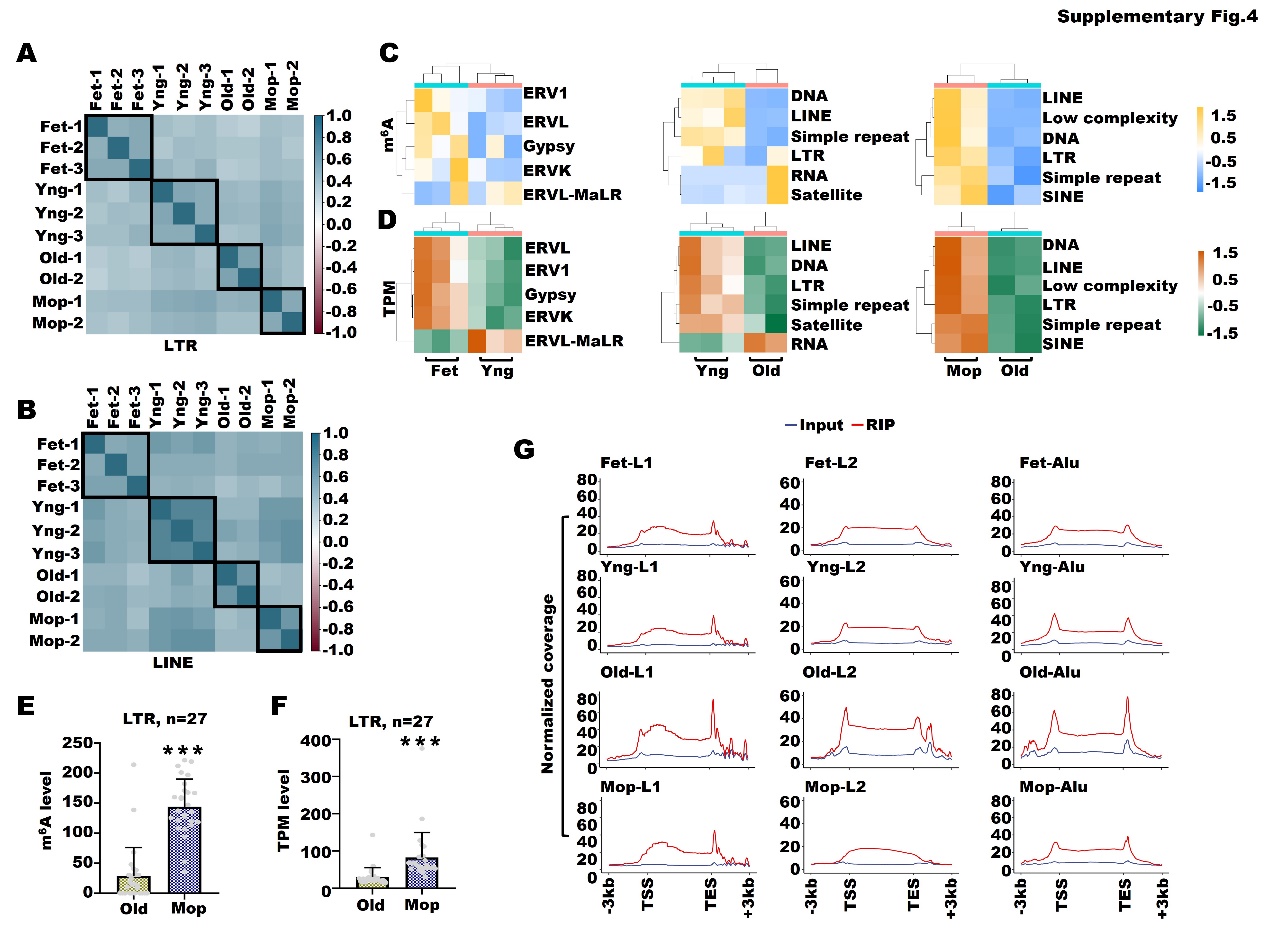


**Fig. S4. The profile of m^6^A methylation and the transcriptome of LTR and non-LTR RNA in four distinct stages, related to Figure 2.**

A-B, Heatmap displaying the Pearson correlation of m^6^A levels in LTR and LINE between matched samples from human ovaries.

C-D, The m^6^A and transcriptome levels of the majority of retrotransposon RNA types during the OD, OFD and OA stages.

E-F, The m^6^A and RNA expression levels of 27 LTR RNAs in Old and Mop.

G, Average profile of m^6^A RIP and input signal of L1, L2 and Alu in Fet, Yng, Old, and Mop. L1=LINE1, L2=LINE2.


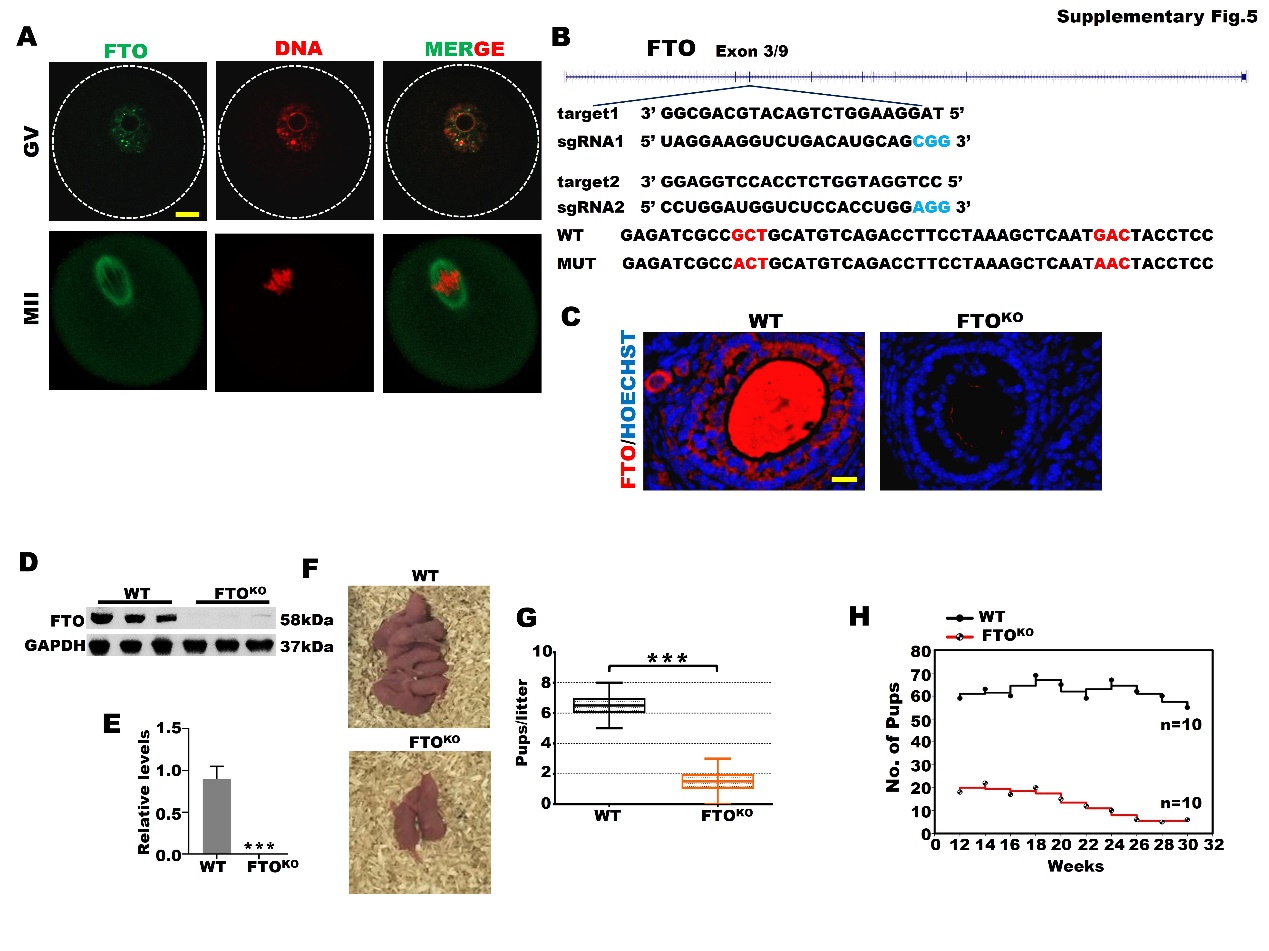


**Fig. S5. FTO^KO^ severely impacted the reproductive capability of mice, related to Figure 3.**

A, Immunofluorescence localization of FTO (green) and nuclear (red) staining in GV and MII oocytes.

B, The design of the CRISPR/Cas9 genome editing system for achieving an FTO knockout in a mouse model.

C, Immunofluorescence localization of FTO (red) and HOECHST (blue) staining in FTO^KO^ and WT oocytes (scale bars, 50 μm).

D-E, Protein expression of FTO in the ovaries of FTO^KO^ and WT mice (****p* < 0.001).

F-G, The number of pups per litter in FTO^KO^ and WT mice from 12 to 24 weeks (****p* < 0.001).

H, The mouse litter number in FTO^KO^ and WT mice from 12 to 30 weeks (n = 10).


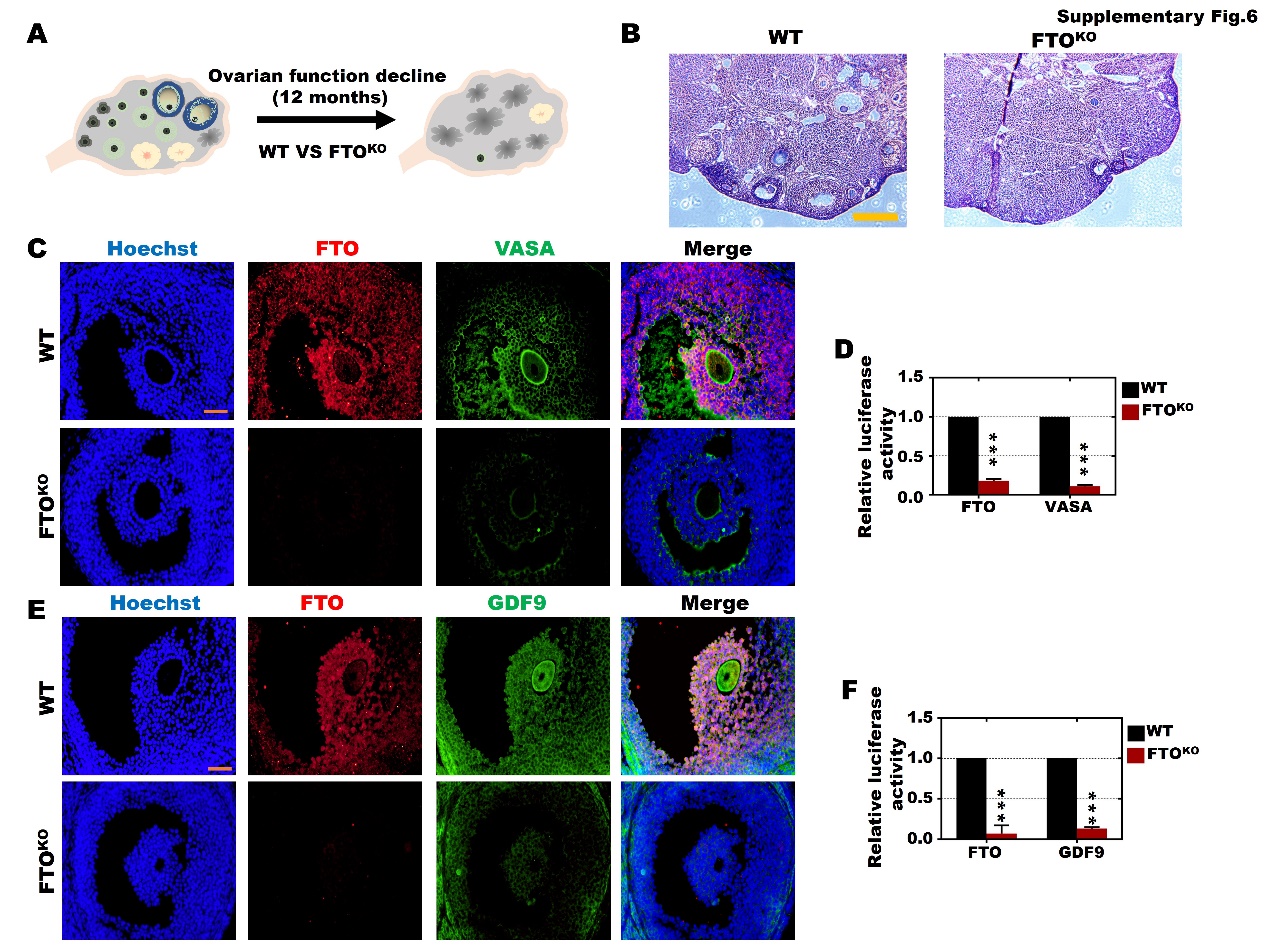


**Fig. S6. Immunofluorescence and HE staining of FTO^KO^ and WT mice at 12 months, related to Figure 3.**

A, Schematic representation of mouse ovaries at 12 months with ovarian functional decline.

B, HE staining of FTO^KO^ and WT mice at 12 months.

C-D, Immunofluorescence of FTO (red) and VASA (green) staining in FTO^KO^ and WT ovaries (scale bars, n = 3, 50 μm, ****p* < 0.001).

E-F, Immunofluorescence of FTO (red) and GDF9 (green) staining in FTO^KO^ and WT ovaries (****p* < 0.001).


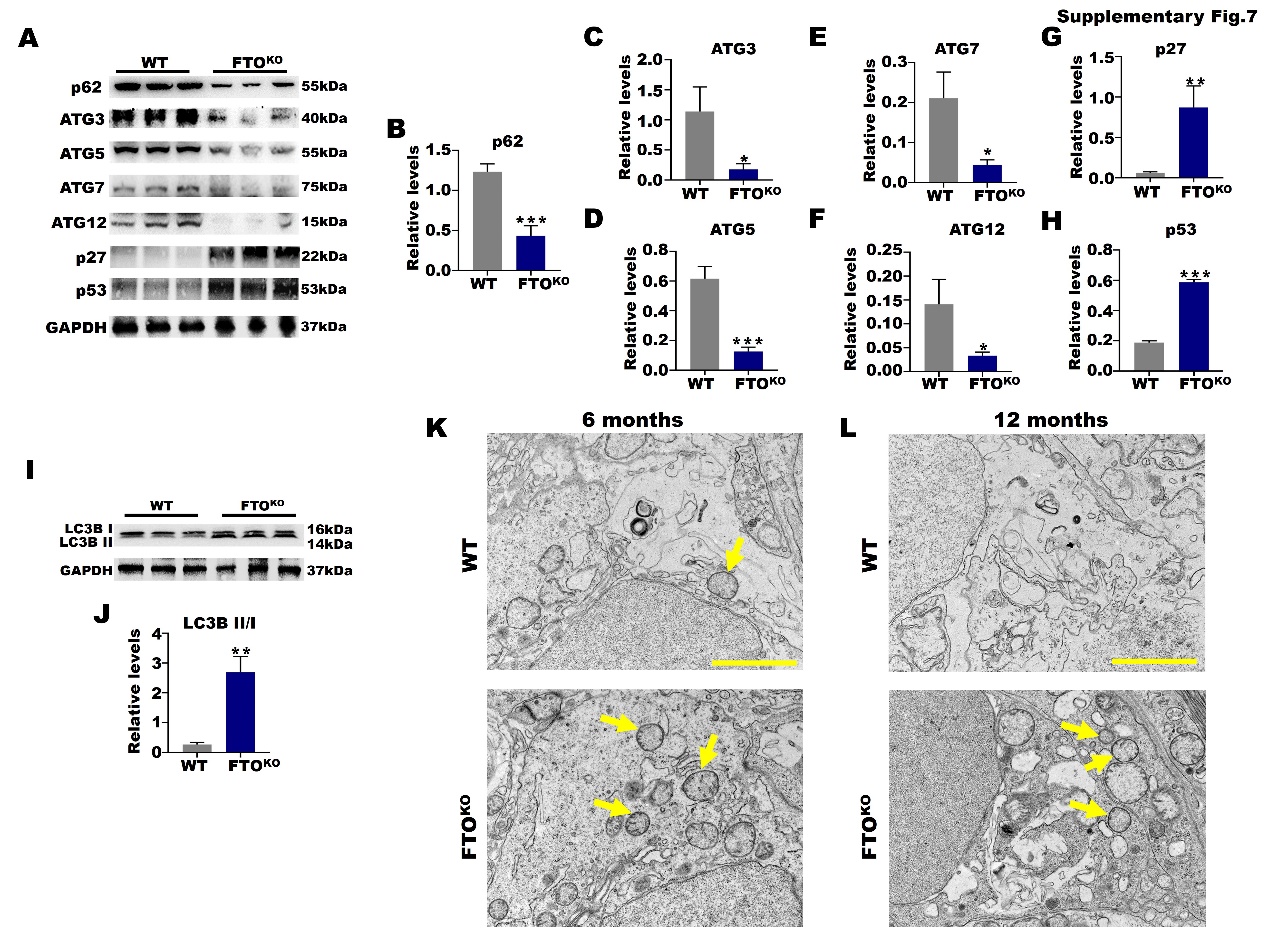


**Fig. S7. FTO^KO^ regulated autophagic flux in mouse ovarian aging, related to Figure 3.**

A-H, Protein expression of autophagy genes (p62, ATG3, ATG5, ATG7, ATG12) and aging genes (p27, and p53) in the ovaries of 6-month-old FTO^KO^ and WT mice (**p* < 0.05, ***p* < 0.01, ****p* < 0.001).

I, The autophagy regulation protein expression of LC3B II and LC3B I in the ovaries of 6-month-old FTO^KO^ and WT mice.

J, The ratio of LC3B II to LC3B I in the ovaries of 6-month-old FTO^KO^ and WT mice (***p* < 0.01).

K-L, Electron microscopy analysis of autophagosomes in the ovaries of 6-month-old and 12-month-old FTO^KO^ and WT mice.


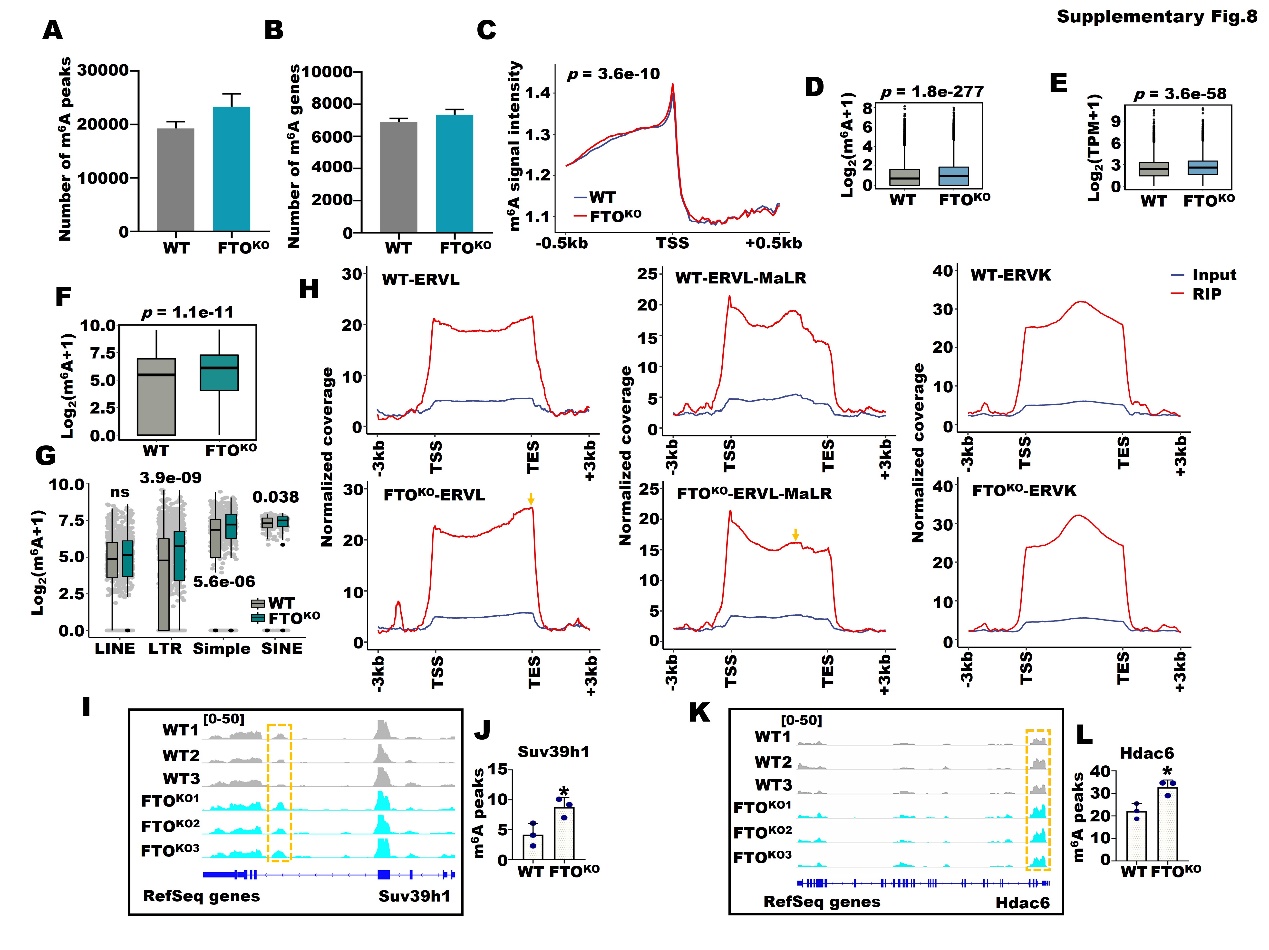


**Fig. S8. FTO^KO^ impacted the overall m^6^A levels of mRNA and specific retrotransposon RNAs as well as histone modification-related adaptors, related to Figure 4.**

A-B, The number of m^6^A peaks and genes in the ovaries of six-month-old FTO^KO^ and WT mice.

C, The m^6^A signal intensity of the total m^6^A genes at the promoter regions in the FTO^KO^ and WT mice.

D-E, Box plot showing the m^6^A and mRNA expression levels of genes in FTO^KO^ and WT mice.

F, Box plot showing the global m^6^A methylation levels of retrotransposon RNA in FTO^KO^ and WT mice.

G, Box plot showing the m^6^A levels of LINEs, LTRs, simple repeats, and SINEs in FTO^KO^ and WT mice.

H, Average profile of m^6^A RIP and input signal of ERVL, ERVL-MaLR and ERVK in FTO^KO^ and WT mice.

I-L, IGV showing the IP and input read distribution of Suv39h1 and Hdac6 across FTO^KO^ and WT mice (**p* < 0.05).


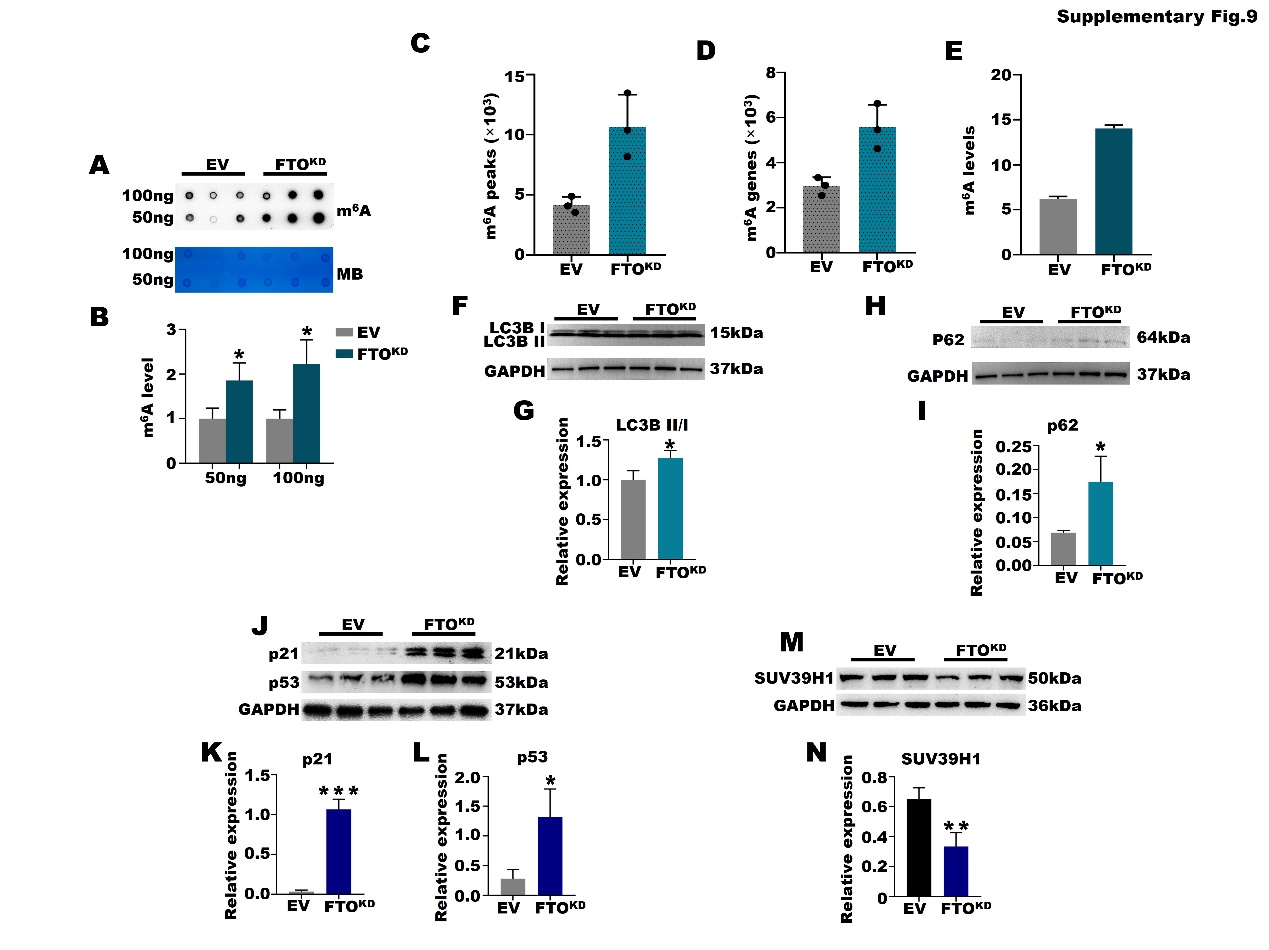


**Fig. S9. FTO^KD^ influenced autophagic flux in the FTO^KD^ KGN cell line, related to Figure 5.**

A-B, The m^6^A dot blot of total RNA samples from FTO^KD^ and EV KGN cell lines.

C-D, The number of m^6^A peaks and genes in the FTO^KD^ and EV KGN cell lines.

E, The m^6^A level of the FTO^KD^ and EV KGN cell lines.

F-G, The protein expression of LC3II/I in the FTO^KD^ and EV KGN cell lines (**p* < 0.05).

H-I, The autophagic protein expression of p62 in the FTO^KD^ and EV KGN cell lines (**p* <0.05).

J-K, The aging protein expression of p21 and p53 in the FTO^KD^ and EV KGN cell lines (**p* <0.05, ****p* <0.001).

M-N, The protein expression of SUV39H1 in the FTO^KD^ and EV KGN cell lines (***p* <0.01).


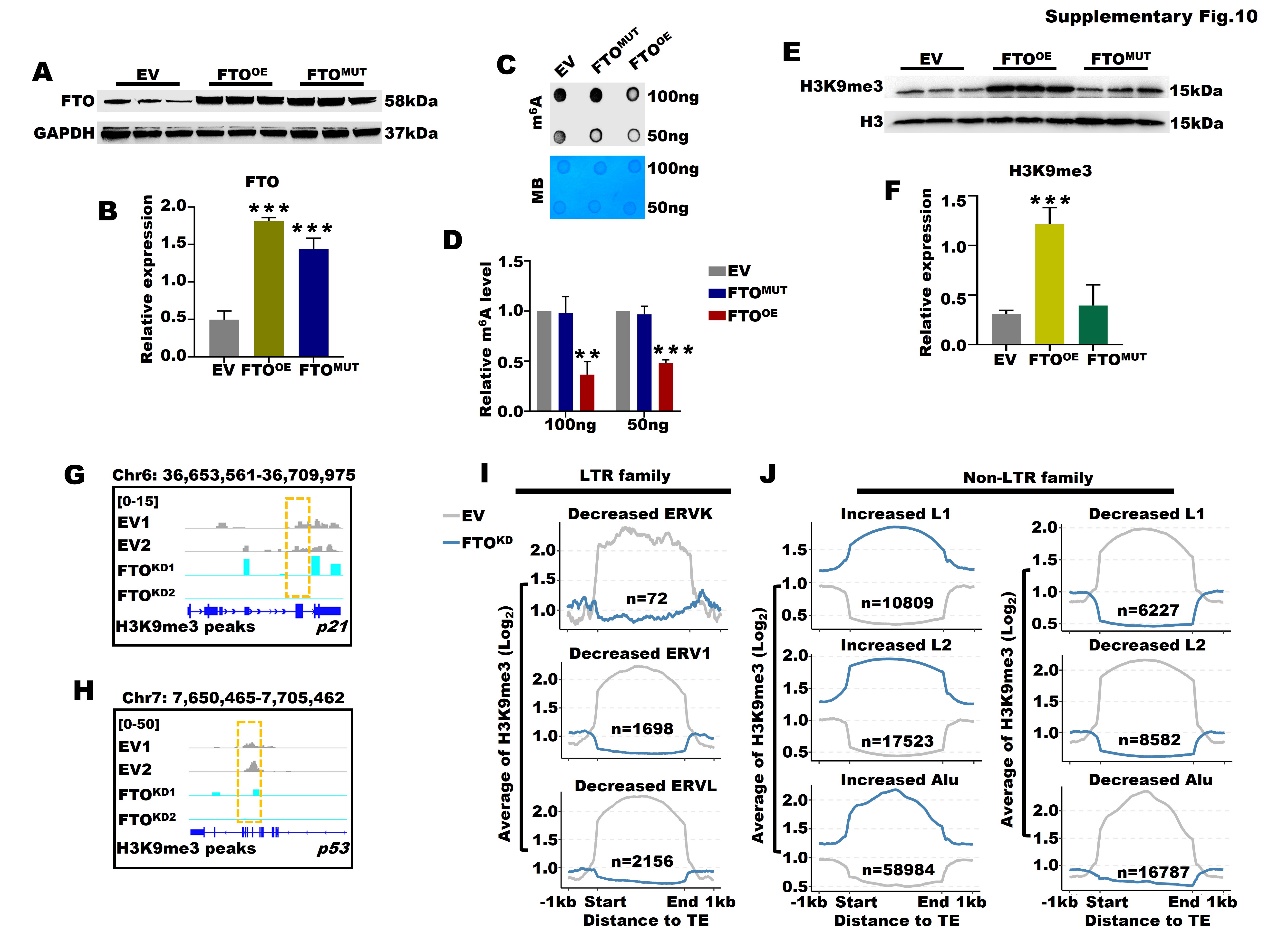


**Fig. S10. FTO^KD^, FTO^OE^, and FTO^MUT^ influenced H3K9me3 on TEs, related to Figure 5.**

A-B, Protein expression of FTO in FTO^OE^ and FTO^MUT^ cell lines (****p* <0.001).

C-D, The m^6^A dot blot of total RNA samples from FTO^OE^ and FTO^MUT^ cell lines. (***p* <0.01, *** *p* <0.001).

E-F, The protein expression of H3K9me3 in FTO^OE^ and FTO^MUT^ cell lines (****p* <0.001).

G-H, H3K9me3 peaks on aging genes (p21 and p53) in the FTO^KD^ and EV cell lines.

I, Average H3K9me3 signal on the decreased ERVK, ERV1, and ERVL in the FTO^KD^ and EV cell lines.

J, Average H3K9me3 signal on the increased and decreased L1, L2, and Alu in the FTO^KD^ and EV cell lines.

**
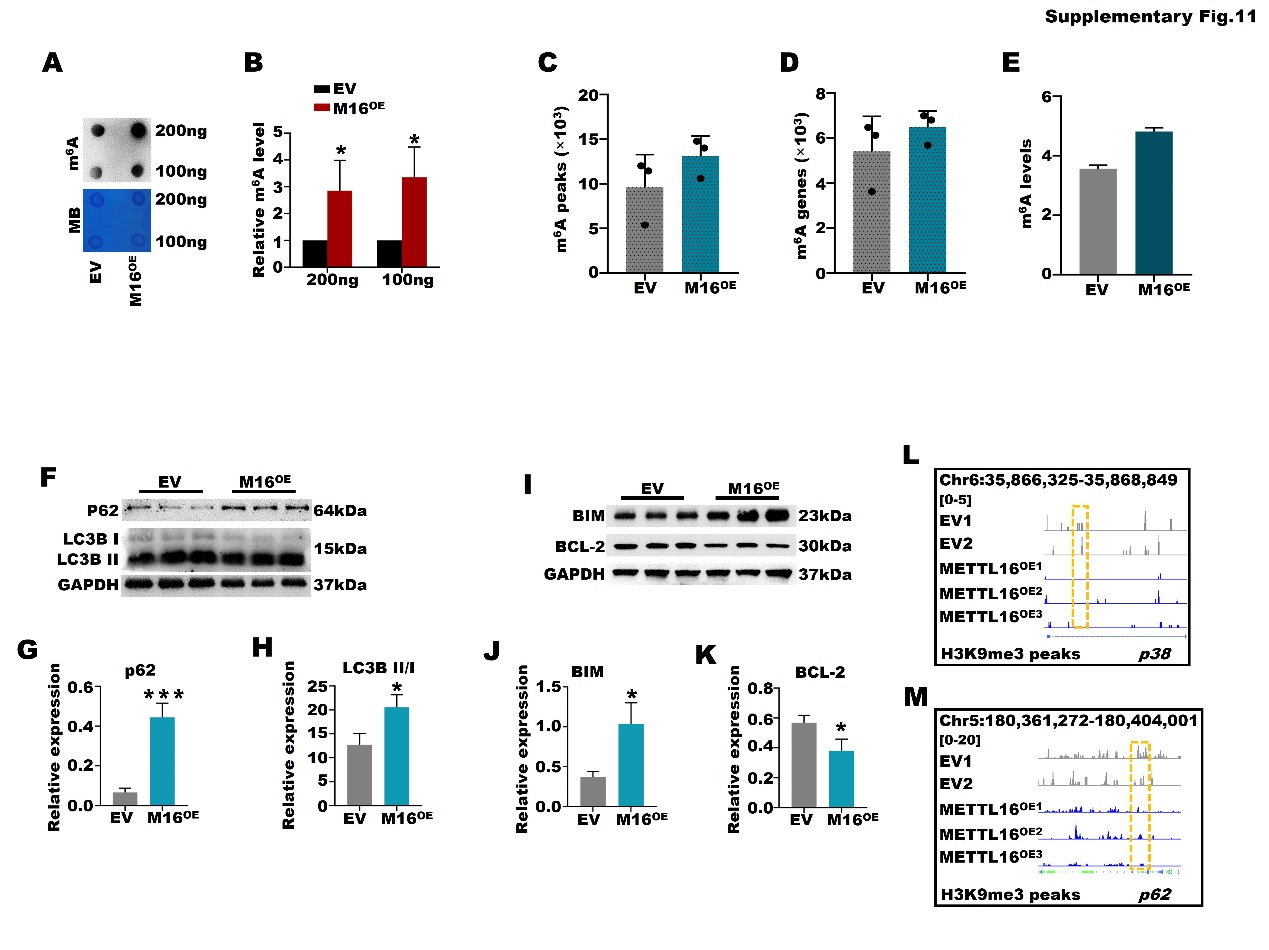
**

**Fig. S11. M16^OE^ influenced autophagic flux in the M16^OE^ KGN cell line, related to Figure 6.**

A-B, The m^6^A dot blot of total RNA samples from the M16 ^OE^ and EV KGN cell lines (**p* < 0.05).

C-D, The number of m^6^A peaks and genes in the M16 ^OE^ and EV KGN cell lines.

E, The m^6^A level of the M16 ^OE^ and EV KGN cell lines.

F-H, Autophagic protein expression (p62 and LC3II/I) in the M16^OE^ and EV KGN cell lines (**p* < 0.05, ****p* <0.001).

I-K, The expression of aging proteins (BIM and BCL-2) in the M16^OE^ and EV KGN cell lines (**p* <0.05).

L-M, H3K9me3 peaks on autophagic (p62) and aging (p38) proteins in the M16^OE^ and EV cell lines.


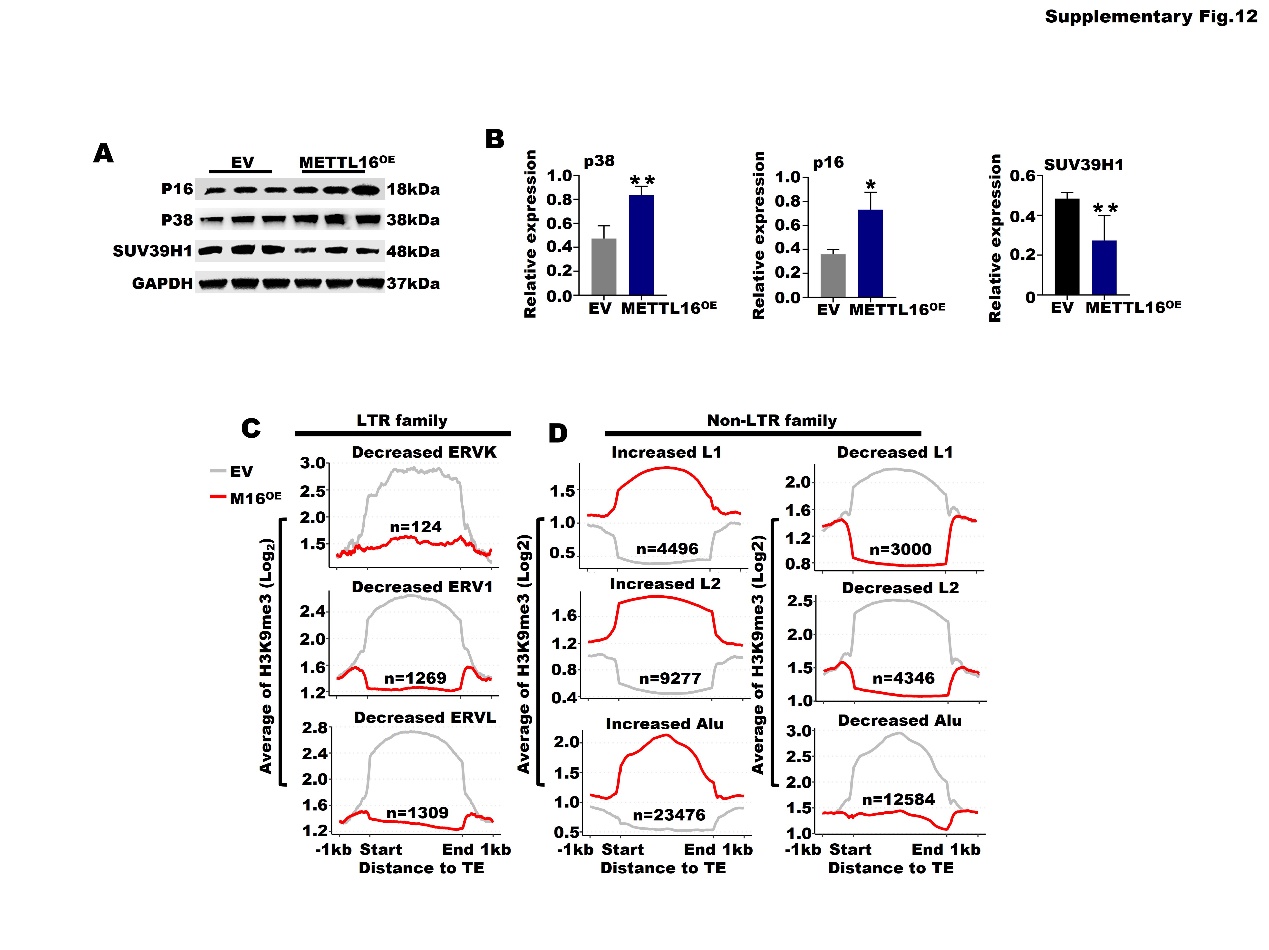


**Fig. S12. M16^OE^ influenced aging genes, SUV39H1 and H3K9me3 on TEs, related to Figure 6.**

A-B, The protein expression of aging genes (p16, p38) and SUV39H1 in the M16^OE^ and EV cell lines (**p* <0.05, ***p* <0.01).

C, Average H3K9me3 signal on the decreased ERVK, ERV1, and ERVL in the M16^OE^ and EV cell lines.

D, Average H3K9me3 signal on the increased and decreased L1, L2, and Alu in the M16^OE^ and EV cell lines.


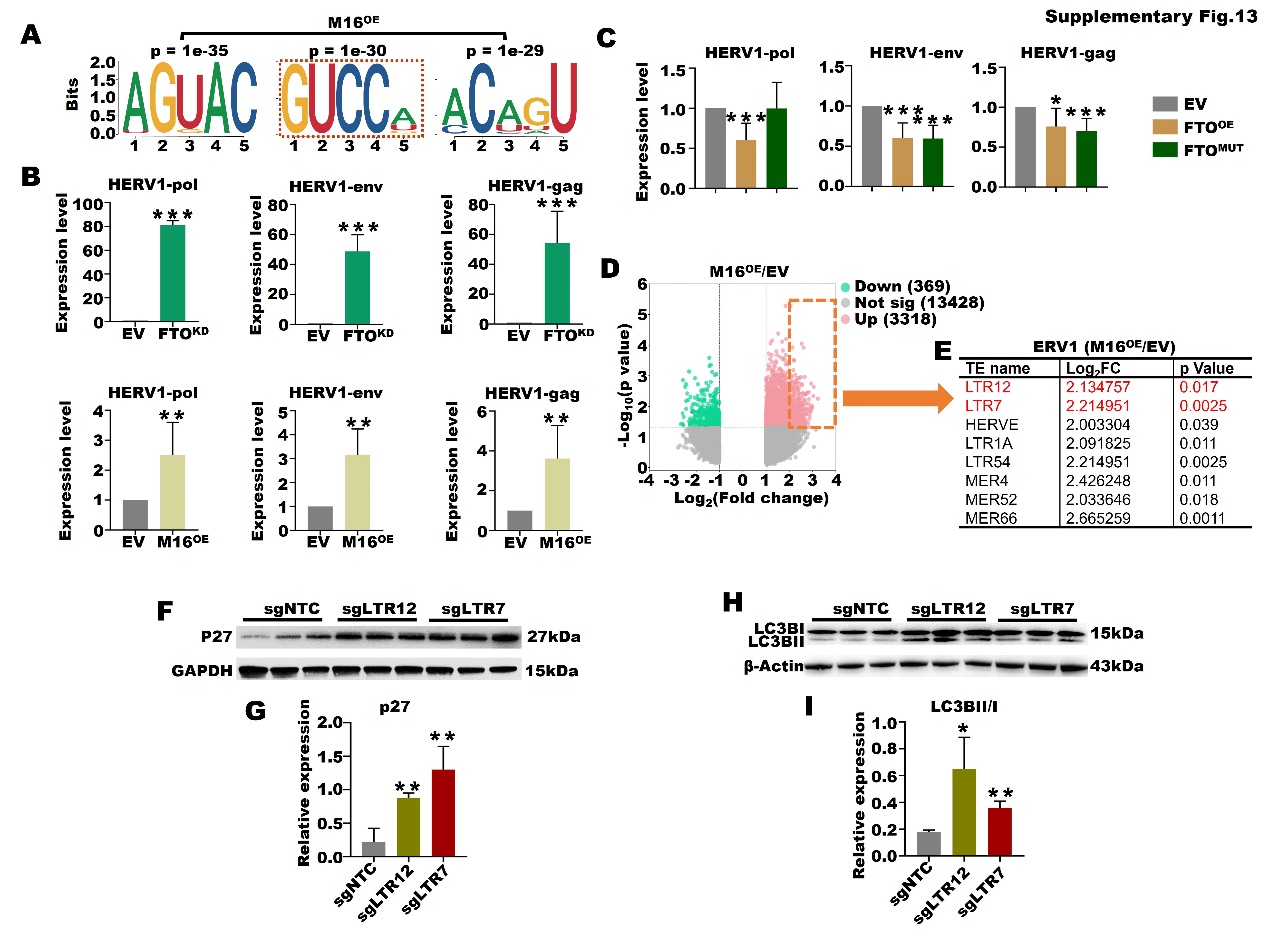


**Fig. S13. The activation of LTR7 and LTR12 induced by high m^6^A methylation levels is a trigger for impairment of autophagy and ovarian aging.**

A, The top three m^6^A motifs in the M16^OE^ groups.

C, The mRNA expression levels of HERV1-pol, HERV1-env and HERV1-gag in the FTO^OE^, FTO^MUT^ and EV cell lines (**p* <0.05, ****p* <0.001).

B, The mRNA expression levels of HERV1-pol, HERV1-env and HERV1-gag in the FTO^KD^ and M16^OE^ cell lines (***p* <0.01, ****p* <0.001).

D, Scatterplots showing differentially expressed TEs in the M16^OE^ group.

E, The eight differentially expressed subfamilies of ERV1 in the M16^OE^ group with FC > 2.

F-G, The aging protein expression of p27 in sgLTR12 and sgLTR7 cells (***p* <0.01).

H-I, Expression of the autophagic protein LC3II/I in sgLTR12 and sgLTR7 cells (**p* <0.05, ***p* <0.01).

Table S1. human ovarian samples information in this study.

| Individual | Age | Tissues Collected |
| --- | --- | --- |
| Fetus_1 | 20 weeks | ovary |
| Fetus_2 | 23 weeks | ovary |
| Fetus_3 | 18 weeks | ovary |
| Young_1 | 25 years old | ovary |
| Young_2 | 26 years old | ovary |
| Young_3 | 30 years old | ovary |
| Old_1 | 46 years old | ovary |
| Old_2 | 45 years old | ovary |
| Mop_1 | 51 years old | ovary |
| Mop_1 | 60 years old | ovary |

Table S2. mouse ovarian samples information in this study.

| Individual | Age | Tissues Collected |
| --- | --- | --- |
| WT_1 | 6 months | ovary |
| WT_2 | 6 months | ovary |
| WT_3 | 6 months | ovary |
| Fto KO_1 | 6 months | ovary |
| Fto KO_2 | 6 months | ovary |
| Fto KO_3 | 6 months | ovary |
| WT_1 | 12 months | ovary |
| WT_2 | 12 months | ovary |
| WT_3 | 12 months | ovary |
| Fto KO_1 | 12 months | ovary |
| Fto KO_2 | 12 months | ovary |
| Fto KO_3 | 12 months | ovary |

Table S3. Designations, sequences, and the sizes of plasmid amplicons

| Name | Sequence from 5'-3' | Size (bp) |
| --- | --- | --- |
| NC (H) | GGTTCTCCGAACGTGTCACGT | 21 |
| FTO KD(H) | ACCTGAACACCAGGCTCTTTA | 21 |
| SgNC (H) | AAGATGAAAGGAAAGGCGTT | 20 |
| SgLTR12F (H) | CTCGCTGGTGCTTACTCTTT | 20 |
| SgLTR7 (H) | TTTGACTGTAATTTTCCACT | 20 |
| FTO empty vector(H) | pcDNA3(Beijing Tsingke Biotech Co., Ltd) |  |
| FTO OE (H) | GenBank Accession No. NP_001073901.1 |  |
| FTO MUT(H) | H231A & D233A |  |
| METLL16 empty(H) | pEnCMV P30743（MiaoLing Biology) |  |
| METLL16 OE(H) | GenBank Accession No.NP_076991.3 |  |

Table S4. Designations, sequences, and the sizes of PCR amplicons

| Name | Sequence from 5'-3' | Size (bp) |
| --- | --- | --- |
| FTO (M) Fw | ccagtgtctcgcatcctcatc | 540 |
| FTO (M) Rev | ttactcatcctcagagcctcaga |  |
| HERV1-gag (H) Fw | ACGCTTTACAGCCCTAGACC | 82 |
| HERV1-gag (H) Rev | GTCGGGAGCAGATTGGGTAA |  |
| HERV1-env(H) Fw | GTATGTCTGATGGGGGTGGAG | 111 |
| HERV1-env(H)Rev | CTAGTCCTTTGTAGGGGCTAGAG |  |
| HERV1-pol(H) Fw | CGCCCTTCTTCCCAATCCAA | 96 |
| HERV1-pol(H) Rev | GCCAAGGAGGGAGTAGAGGT |  |
| β-Actin (H) Fw | TCCCTGGAGAAGAGCTACGA | 193 |
| β-Actin (H) Rev | AGCACTGTGTTGGCGTACAG |  |

H=Human; M=Mouse.

Table S5 KEY RESOURCES

| REAGENTor RESOURCE | SOURCE | IDENTIFIER |
| --- | --- | --- |
| Rabbit anti-m6A | Cell Signaling Technology | Cat# 56593S; RRID: AB_2799515 |
| Mouse anti-FTO | Millipore | Cat# MABE227; RRID: AB_11203491 |
| Rabbit anti-FTO | ProteinTech | Cat# 27226-1-AP; RRID:AB_2880809 |
| Rabbit anti-VASA | Abcam | Cat# AB13840; RRID: AB_443012 |
| Goat anti-GDF9 | R and D Systems | Cat# AF739; RRID: AB_2111517 |
| Mouse Anti-H2A.X | Abcam | Cat# AB26350; RRID: AB_470861 |
| Rabbit anti-Caspase3 | ProteinTech | Cat#19677-1AP; RRID:AB_10733244 |
| Rabbit anti-Caspase9 | Abcam | Cat# AB202068; RRID: AB_2889070 |
| Rabbit anti-SUV39H1 | Cell Signaling Technology | Cat# 8729; RRID: AB_10829612 |
| Rabbit anti-H3K9me3 | Abcam | Cat# ab176916; RRID: 25614-1-AP |
| Rabbit anti-H3 | ProteinTech | Cat# 17168-1-AP; RRID: AB_2716755 |
| Rabbit anti-Laminb1 | ProteinTech | Cat# 12987-1-AP; RRID: AB_2136290 |
| Rabbit anti-P21 | ProteinTech | Cat# 10355-1-AP; RRID: AB_2077682 |
| Rabbit anti-P16 | ProteinTech | Cat# 10883-1-AP; RRID: AB_2078303 |
| Rabbit anti- P27 | ProteinTech | Cat# 25614-1-AP; RRID: AB_2880161 |
| Rabbit anti- P38 | ProteinTech | Cat# 14064-1-AP; RRID: AB_2878007 |
| Mouse anti-P53 | ProteinTech | Cat# 60283-2-Ig; RRID: AB_2881401 |
| Rabbit anti-P62 | Abcam | Cat# ab109012; RRID: AB_2810880 |
| Mouse anti-METTL16 | Novus | Cat# NBP2-02026; RRID: AB_2724677 |
| Rabbit anti-LC3B | Abcam | Cat# ab51520; RRID: AB_881429 |
| Rabbit anti-BIM | Cell Signaling Technology | Cat# 2933; RRID: AB_1030947 |
| Rabbit anti-BCL-2 | Abcam | Cat# AB182858; RRID: AB182858 |
| Rabbit anti-ATG3 | Cell Signaling Technology | Cat# 3415; RRID: AB_2059244 |
| Rabbit anti-ATG5 | Abcam | Cat# AB108327; RRID: AB_2650499 |
| Rabbit anti-ATG7 | Cell Signaling Technology | Cat# 8558; RRID: AB_10831194 |
| Rabbit anti-ATG12 | Cell Signaling Technology | Cat# 4180; RRID: AB_1903898 |
| Rabbit anti-GAPDH | ProteinTech | Cat# 10494-1-AP; RRID: AB_2263076 |
| Mouse anti-β-Actin | ProteinTech | Cat# 66009-1-Ig; RRID: AB_2687938 |
| Rabbit anti-Ki67 | Abcam | Cat# AB15580; RRID: AB_443209 |
| Alexa Fluor 488 anti-Mouse | ThermoFisher | Cat# A21202; RRID: AB_141607 |
| Alexa Fluor 555 anti-Rabbit | ThermoFisher | Cat# A31572; RRID: AB_162543 |
| HRP*Goat Anti RabbitIgG(H+L) | Immnoway | Cat# RS0002; |
| HRP* Goat Anti Mouse IgG(H+L) | Immnoway | Cat# RS0001; |
| Dylight 488, Rabbit Anti-Goat | Abbkine | Cat# A23230; |

Table S6 Key reagent

| REAGENT or RESOURCE | SOURCE | IDENTIFIER |
| --- | --- | --- |
| DMEM Media | Gibco | Cat# 11995065 |
| Fetal bovine serum | Gibco | Cat# 10099141 |
| Bovine Serum Albumin (BSA) | Sigma | Cat# B2064; CAS# 9048-46-8 |
| Penicillin streptomycin - | Gibco | Cat# 15140122 |
| Triton X-100 | Sigma | Cat# 93443; CAS# 9036-19-5 |
| Hoechst 33342 | Beyotime | Cat# C1025 |
| DAPI | Beyotime | Cat# C1005 |
| M2 Media | Sigma | Cat# M7167 |
| Pregnant Mare’s Serum Gonadotropin (PMSG) | Nanjing Aibei | Cat# M2620 |
| Lipofectamin 2000 | Invitroge | Cat# 11668019 |
| Skim milk | BD | Cat# 232100 |
| Hybond N+ membranes | GE Healthcare | Cat# RPN303C |
| PVDF membrane | Millipore | Cat# IPVH00010 |
| protease inhibitor | Roche | Cat#11873580001 |
| protein A Dynabeads | Thermo Scientific | Cat# 10002D |
| protein G Dynabeads | Thermo Scientific | Cat# 10004D |
| Qubit RNA HS Assay | Thermo Scientific | Cat# Q32852 |
| Equalbit 1x dsDNA HS Assay Kit EQ121 | Vazyme | Cat# EQ121-02 |
| NP-40 Surfact-Amps | Thermo Scientific | Cat# 28324 |
| CUT&Tag Kit | Vazyme | Cat# TD903-01 |
| The EU nascent RNA Detection kit | RIBOBIO | Cat# C10316-1 |
| The EU nascent RNA Detection kit | RIBOBIO | Cat# C10316-3 |
| RNAiso Plus | Takara | Cat# 9109 |
| SYBR premix | BioSharp | Cat# 9109 |
| Turbo DNAase | Invitgen | Cat# BL698A |

Dataset S1 (separate file). A summary of MeRIP sequencing statistics for each sample.

| Individual | Mapped  MeRIP-Seq Reads | Mapped  Input-Seq Reads | MeRIP-Seq  Accessions | Input-Seq  Accessions |
| --- | --- | --- | --- | --- |
| Fetus_1 | 35867205 | 38585801 | HRR1376531 | HRR1376541 |
| Fetus_2 | 30309848 | 34290168 | HRR1376532 | HRR1376542 |
| Fetus_3 | 28978467 | 36570625 | HRR1376533 | HRR1376543 |
| Young_1 | 29512597 | 29909837 | HRR1376534 | HRR1376544 |
| Young_2 | 35415019 | 44120928 | HRR1376535 | HRR1376545 |
| Young_3 | 40684216 | 38858316 | HRR1376536 | HRR1376546 |
| Old_1 | 36722365 | 30567128 | HRR1376537 | HRR1376547 |
| Old_2 | 40177678 | 32410414 | HRR1376538 | HRR1376548 |
| Mop_1 | 51058252 | 44140004 | HRR1376539 | HRR1376549 |
| Mop_2 | 50242359 | 36089273 | HRR1376540 | HRR1376550 |

Software S1 (separate file). Software and algorithms

| SOFTWARE | SOURCE | IDENTIFIER |
| --- | --- | --- |
| Software and algorithms |  |  |
| Prism 9 version 9.2.0 | GraphPad | https://www.graphpad.com/scientificsoftware/prism/ |
| ImageJ | NIH | https://imagej.nih.gov/ij/ |
| Python 3.9 | Python | https://www.python.org/ |
| R | The R Project for Statistical Computing | https://www.r-project.org/ |
| Bowtie 2.4.1 | Langmead et al. ^1^ | https://bowtie-bio.sourceforge.net/ |
| HISAT2 2.2.1 | Kim et al. ^2^ | http://daehwankimlab.github.io/hisat2/ |
| BEDTools 2.29.2 | BEDTools | https://bedtools.readthedocs.io/en/latest/ |
| Trimmomatic 0.36 | Bolger et al.^3^ | http://www.usadellab.org/cms/?page=trimmomatic |
| MACS2 2.2.7 | Zhang et al. ^4^ | <https://pypi.python.org/pypi/MACS2> |
| IGV software (version 2.4.15) | Robinson et al. ^5^ | https://software.broadinstitute.org/software/igv/ |
| STAR 2.7.8a | Dobin et al. ^6^ | https://github.com/alexdobin/STAR |
| FeatureCounts 2.0.1 | Liao et al. ^7^ | https://subread.sourceforge.net/featureCounts.html |
| FindMotifsGenome.pl script | HOMER | http://homer.ucsd.edu/homer/motif |

**SI References**

1. Langmead, B., and Salzberg, S.L. (2012). Fast gapped-read alignment with Bowtie 2. Nat Methods *9*, 357-359. 10.1038/nmeth.1923.

2. Kim, D., Paggi, J.M., Park, C., Bennett, C., and Salzberg, S.L. (2019). Graph-based genome alignment and genotyping with HISAT2 and HISAT-genotype. Nat Biotechnol *37*, 907-915. 10.1038/s41587-019-0201-4.

3. Bolger, A.M., Lohse, M., and Usadel, B. (2014). Trimmomatic: a flexible trimmer for Illumina sequence data. Bioinformatics *30*, 2114-2120. 10.1093/bioinformatics/btu170.

4. Zhang, Y., Liu, T., Meyer, C.A., Eeckhoute, J., Johnson, D.S., Bernstein, B.E., Nusbaum, C., Myers, R.M., Brown, M., Li, W., and Liu, X.S. (2008). Model-based analysis of ChIP-Seq (MACS). Genome Biol *9*, R137. 10.1186/gb-2008-9-9-r137.

5. Robinson, J.T., Thorvaldsdottir, H., Winckler, W., Guttman, M., Lander, E.S., Getz, G., and Mesirov, J.P. (2011). Integrative genomics viewer. Nat Biotechnol *29*, 24-26. 10.1038/nbt.1754.

6. Dobin, A., Davis, C.A., Schlesinger, F., Drenkow, J., Zaleski, C., Jha, S., Batut, P., Chaisson, M., and Gingeras, T.R. (2013). STAR: ultrafast universal RNA-seq aligner. Bioinformatics *29*, 15-21. 10.1093/bioinformatics/bts635.

7. Liao, Y., Smyth, G.K., and Shi, W. (2014). featureCounts: an efficient general purpose program for assigning sequence reads to genomic features. Bioinformatics *30*, 923-930. 10.1093/bioinformatics/btt656.

**Abbreviation:**

N6-methyladenosine: m^6^A, fat mass and obesity-associated protein: FTO, methyltransferases-like protein 16: METTL16, endogenous retroviruses: ERVs, long terminal repeat retrotransposons: LTR, Gene Ontology: GO, transcription start sites: TSSs, transcription end sites: TESs, wild type: WT, empty vector: EV, transposable elements: TEs, knockout: KO, knockdown: KD, overexpression: OE, methylated RNA immunoprecipitation sequencing: MeRIP, fetal: Fet, young: Yng, menopausal: Mop, m^6^A-positive: m^6^A+, m^6^A-negative: m^6^A-, ovarian development: OD, ovarian function decline: OFD, ovarian aging: OA, immunofluorescence: IF, N6-2-O-dimethyladenosine: m^6^Am, N1-methyladenosine: m^1^A, human endogenous retrovirus 1: HERV1, histone H3 containing the trimethylated lysine 9: H3K9me3, suppressor of variegation 3-9 homolog 1: SUV39H1, suppressor of variegation 4-0 homolog 2: SUV420H2.
